# Detection and visualization of communities in mass spectrometry imaging data

**DOI:** 10.1186/s12859-019-2890-6

**Published:** 2019-06-04

**Authors:** Karsten Wüllems, Jan Kölling, Hanna Bednarz, Karsten Niehaus, Volkmar H. Hans, Tim W. Nattkemper

**Affiliations:** 1 International Research Training Group “Computational Methods for the Analysis of the Diversity and Dynamics of Genomes”, Bielefeld University, Universitätsstraße 25, Bielefeld, 33613 Germany; 2 Biodata Mining Group, Faculty of Technology, Bielefeld University, Universitätsstraße 25, Bielefeld, 33613 Germany; 3 Center for Biotechnology (CeBiTec), Universitätsstraße 25, Bielefeld, 33613 Germany; 4 Proteome and Metabolome Research, Faculty of Biology, Bielefeld University, Universitätsstraße 25, Bielefeld, 33613 Germany; 5 Department of Neuropathology, Institute for Clinical Pathology, Dietrich-Bonhoeffer-Klinikum, Salvador-Allende-Straße 30, Neubrandenburg, 17036 Germany; 6 Department of Neuropathology, Essen University Hospital (AöR), Hufelandstraße 55, Essen, 45147 Germany

**Keywords:** MALDI imaging, Networks, Clustering, Community detection, Visualization, Graphs

## Abstract

**Background:**

The spatial distribution and colocalization of functionally related metabolites is analysed in order to investigate the spatial (and functional) aspects of molecular networks. We propose to consider community detection for the analysis of *m/z*-images to group molecules with correlative spatial distribution into communities so they hint at functional networks or pathway activity. To detect communities, we investigate a spectral approach by optimizing the modularity measure. We present an analysis pipeline and an online interactive visualization tool to facilitate explorative analysis of the results. The approach is illustrated with synthetical benchmark data and two real world data sets (barley seed and glioblastoma section).

**Results:**

For the barley sample data set, our approach is able to reproduce the findings of a previous work that identified groups of molecules with distributions that correlate with anatomical structures of the barley seed. The analysis of glioblastoma section data revealed that some molecular compositions are locally focused, indicating the existence of a meaningful separation in at least two areas. This result is in line with the prior histological knowledge. In addition to confirming prior findings, the resulting graph structures revealed new subcommunities of *m/z*-images (i.e. metabolites) with more detailed distribution patterns. Another result of our work is the development of an interactive webtool called GRINE (Analysis of **GR**aph mapped **I**mage Data **NE**tworks).

**Conclusions:**

The proposed method was successfully applied to identify molecular communities of laterally co-localized molecules. For both application examples, the detected communities showed inherent substructures that could easily be investigated with the proposed visualization tool. This shows the potential of this approach as a complementary addition to pixel clustering methods.

**Electronic supplementary material:**

The online version of this article (10.1186/s12859-019-2890-6) contains supplementary material, which is available to authorized users.

## Introduction

Matrix-assisted laser desorption ionization mass spectrometry imaging (MALDI-MSI) is a rapidly developing technology for investigating the lateral distribution of molecules in biological samples in form of multivariate bioimages [1].

Due to the technological improvements and the increased utilization of MALDI-MSI, the daily amount of generated data is constantly increasing [2]. Since the complete interpretation cannot be automated, semi-automated and assistive computational methods appear promising and are in the focus of our research.

Different methods for grouping MSI data have already been investigated for the analysis of MSI data, such as: k-means [3], hierarchical clustering [4], hierarchical hyperbolic self-organizing maps [5], high dimensional discriminant clustering [6], or probabilistic latent semantic analysis [7]. Many of these studies focus on clustering of all spectra in one data set to achieve a segmentation map, i.e. the partition of the image into regions with high intrinsic spectra similarity [5, 6]. In other words: most approaches focus on spectral similarity to group pixels.

The approach presented in this paper focuses on the grouping of molecules into molecular communities. We assume that many functionally related molecules may feature a similar lateral distribution in the sample. Thus, our method groups molecules into communities based on the similarity of their *m/z*-images. Graphs are well known data structures in biology. Therefore, we propose to use community detection for grouping [8, 9], also known as graph clustering. In our approach, one graph represents one MSI data set of *N*_V_*m/z*-images. The *N*_V_*m/z*-images are usually selected by a user and/or an automated selection of *N*_V_ peaks. A node *v*_*i*_ of the graph corresponds to one *m/z*-image $I_{(m/z)_{i}}$, with *i*∈1,…,*N*_V_, where:

*N*_V_=*#*nodes and *#*nodes=*#m*/*z*-images.

**Fig. 1:**
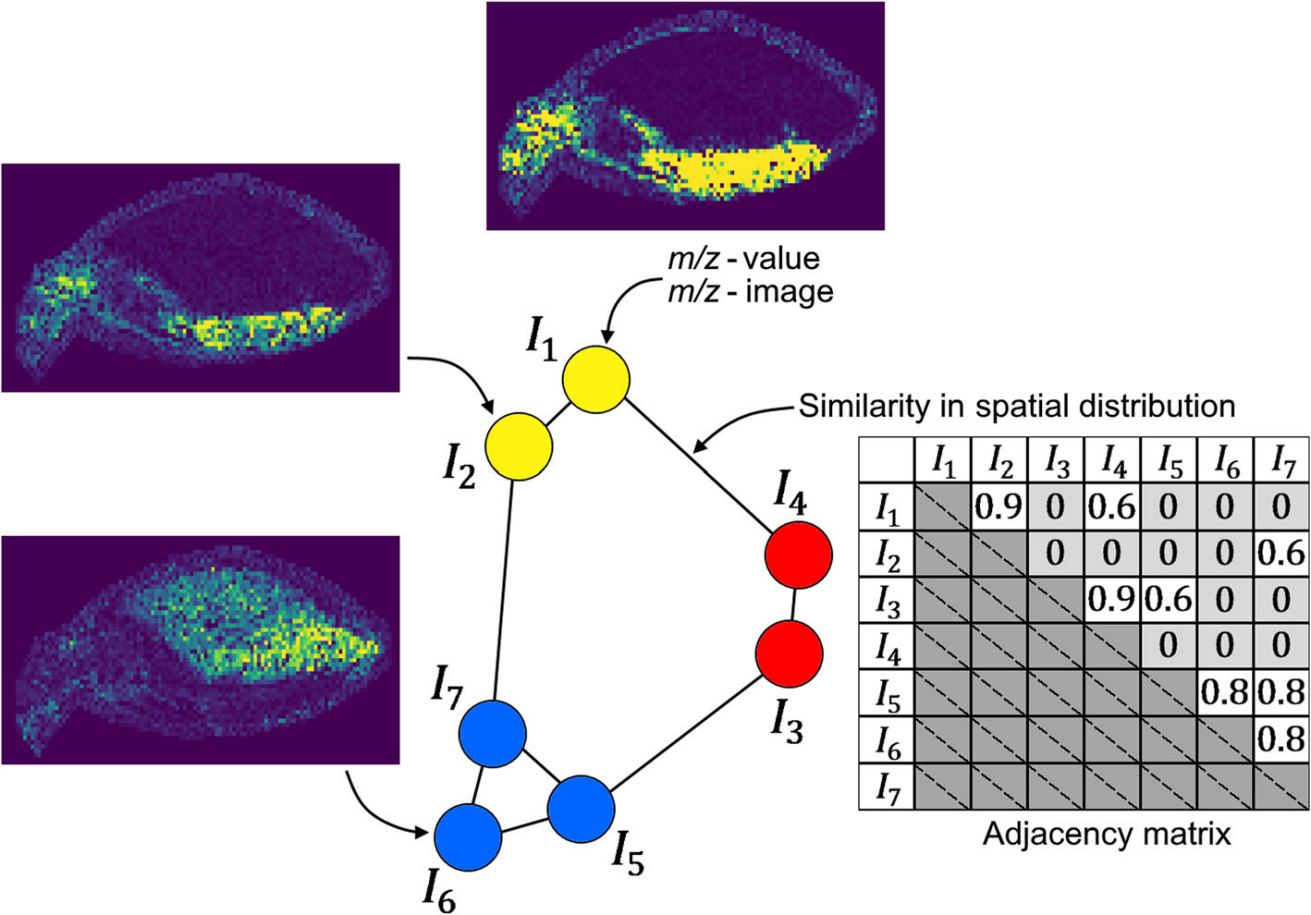
Structure of the *m/z*-image similarity graph. Each node represents an *m/z*-image, each edge represents the similarity between the *m/z*-images it connects, requiring that this value is above a specific threshold. Each color encodes one community

Each edge *e*_*k*_={*v*_*i*_,*v*_*j*_}, with:

$$\begin{array}{*{20}l} &k \in 1, \dots,N_{\mathrm{E}}\ \text{and}\ i,j \in 1, \dots, N_{\mathrm{V}}, \text{ where:}\\ &N_{\mathrm{E}}=\#\text{edges} \end{array} $$

has a weight *w*_*ij*_, which represents the similarity of the spatial signal distribution:

1 $$ w_{i,j} = \text{similarity}(I_{(m/z)_{i}}, I_{(m/z)_{j}})   $$

between the *m/z*-images of nodes *v*_*i*_ and *v*_*j*_. In its initial form the graph is fully connected. Our goal is to identify communities of similar spatial distribution in order to identify groups of functionally related molecules. The method is illustrated in Fig. 1 for a hypothetical data set of *N*_V_=7 images and an adjacency matrix leading to three communities.

To the best of our knowledge, community detection is a new approach for MALDI-MSI data. It provides an uncommon view on the data as we focus on groups of similar spatial distributions rather than spectra similarity (pixel similarity). Few previous works have already shown the benefit of the analysis of spatial distributions in MSI ([10, 11]). Moreover, our approach provides a graph structure that serves as an additional source of information.

To tackle the problem of finding communities of *m/z*-images featuring a similar spatial signal distribution, we developed a modular analysis pipeline consisting of five major blocks : 1. data preprocessing, 2. computation of a *N*_V_×*N*_V_ similarity matrix **S**, 3. transforming the similarity matrix into an *N*_V_×*N*_V_ adjacency matrix **A**, 4. community detection and 5. interactive visualization. Step 5 aims to obtain additional information from the graph that is not available through the community detection result itself.

## Methods

### Data sets

MALDI-MSI data forms a three dimensional data cube, where the *x*–axis and the *y*–axis represent the lateral coordinates (pixels), which can be represented as intensity images also called *m/z*-images, while the *z*–axis represents the mass spectra information. In this study three data sets are used. The first one is a synthetical benchmark data set and consists of nine generated 2*D* gaussians (*D*_G_) (please find details below), the second data set (*D*_B_) was gathered from a germinating barley seed timeline experiment [12] and the third one (*D*_T_) was recorded from a section of a human glioblastoma tumor [13]. *D*_B_ and *D*_T_ are in-house produced data sets.

**Fig. 2:**
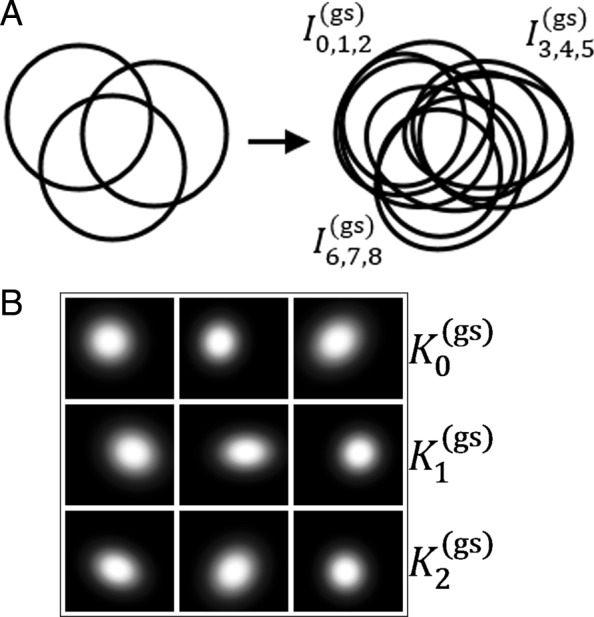
**a** A sketch of how the groups of 2*D* - gaussians are located (left) and how they are distorted (right). **b** The nine rendered 2*D*-gaussian distribution images

*D*_G_ consists of nine synthetic *m/z*-images $I^{\text {(gs)}}_{0} \dots I^{\text {(gs)}}_{8}$ and is a synthetical 9×205×190 MSI toy data cube. Each image contains a single localized 2*D* gaussian intensity distribution. The gaussians were initialized with the same size, a slightly different amplitude and were placed in groups of three:

$$\begin{array}{*{20}l} &K^{\text{(gs)}}_{0} = \left\{I^{\text{(gs)}}_{0}\, I^{\text{(gs)}}_{1}\, I^{\text{(gs)}}_{2}\right\},\\ &K^{\text{(gs)}}_{1} = \left\{I^{\text{(gs)}}_{3}\, I^{\text{(gs)}}_{4}\, I^{\text{(gs)}}_{5}\right\},\\ &K^{\text{(gs)}}_{2} = \left\{I^{\text{(gs)}}_{6}\, I^{\text{(gs)}}_{7}\, I^{\text{(gs)}}_{8}\right\} \end{array} $$

at three different spatial locations $L^{\text {(gs)}}_{0}$, $L^{\text {(gs)}}_{1}$, $L^{\text {(gs)}}_{2}$, respectively. The placement is made in such a way that it is ensured that the three groups overlap with each other in all possible combinations. This is followed by a small random distortion of the position, *x* size and *y* size, combined with a randomized rotation. A sketch of the gaussians and their variation is shown in Fig. 2.

If we think of a biological analogy for this experiment, each distorted gaussian represents the distribution of a different molecule. Each location $L^{\text {gs}}_{i}$, with *i*=0,1,2, represents the area of a spatially bound metabolic process referred to as pseudo-network, meaning that the molecules distributed in this area are likely to take part in this process.

The original data output of *D*_B_ and *D*_T_ were transformed to the form: *D*=*N*_P_×*N*_V_, where *N*_V_ is the dimension of vector $\mathbf {x} \in \mathbb {R}^{N_{\mathrm {V}}}$, representing the spectrum information and *N*_P_ is the dimension of vector $\mathbf {p} \in \mathbb {N}^{(m \times n)}$ with *m* and *n* are width and height of the visual field, representing the lateral information. To be more precise, the elements of **p** include only the measuring coordinates of the MALDI procedure, i.e. pixel grid cells. Regarding the rendered *m/z*-image, (*x*_*i*_,*y*_*j*_) are pixels matching the area of the measured sample. Furthermore, in our data sets the mass spectra information $\mathbf {x} = (x_{0}, \dots, x_{N_{\mathrm {V}}-1})$, called *m/z*-feature vector, does not represent the whole originally measured spectra, since a set of *N*_V_ interesting *m/z*-values were pre-selected by three of the authors (MG, HB, KN) based on their tissue specific and non-homogenous distribution within the tissue section. Applied to *D*_B_ and *D*_T_ this results in a dimensionality of:

$$\begin{array}{*{20}l} &D_{\mathrm{B}} = N_{\mathrm{P}}^{(2)} \times N_{\mathrm{V}}^{(2)} = 3422 \times 101 \text{ and}\\ &D_{\mathrm{T}} = N_{\mathrm{P}}^{(3)} \times N_{\mathrm{V}}^{(3)} = 28684 \times 106\text{.} \end{array} $$

The preprocessing finishes with winsorizing the upper 1% of intensities for each image:

$$x_{l}= \begin{cases} Q_{99}(x_{l}), & \text{if}\ x_{l} > Q_{99}(x_{l})\text{,} \forall l \in [0, \dots, N_{\mathrm{V}}-1]\\ x_{l}, & \text{otherwise} \end{cases} $$

where *Q*_99_ is the 99*th* quantile.

### Analysis pipeline

To compute the similarity matrix **S** we propose to apply the Pearson correlation coefficient:

2 $$ w_{ij} = \frac{\text{cov}(\mathbf{p}_{i},\mathbf{p}_{j})}{\sigma_{\mathbf{p}_{i}} \sigma_{\mathbf{p}_{j}}}   $$

where cov(**p**_*i*_,**p**_*j*_) is the covariance of the intensity images **p**_*i*_, **p**_*j*_ of the nodes (i.e. metabolites) *v*_*i*_, *v*_*j*_ and $\sigma _{\mathbf {p}_{i}}\phantom {\dot {i}\!}$, $\sigma _{\mathbf {p}_{j}}\phantom {\dot {i}\!}$ are the standard deviations of **p**_*i*_, **p**_*j*_, respectively. The Pearson correlation coefficient is a commonly used similarity measure in the area of MALDI imaging analysis [14–17] and provides a straight forward interpretation. The result is a similarity matrix **S**, with *S*_*i*,*j*_=*w*_*ij*_. Please note that also other symmetric similarity measures can be applied here, such as mutual information or cosine similarity. For more information about considered alternatives we would like to refer the interested reader to S17 of the Additional file 1.

Next, we transform the similarity matrix into an adjacency matrix (step 3) **S**→**A**, where **A** is a much sparser adjacency matrix by thresholding with *t*_S_:

$$A_{i,j} = \begin{cases} 0, & \text{if}\ w_{ij} < t_{\mathrm{S}} \\ 1, & \text{otherwise} \end{cases} $$

The objective is to filter out edges with values too low, so that we can assume that these are unlikely to represent a biologically relevant similarity. However, the selection of *t*_S_ is a non-trivial task. To avoid time consuming manual tuning we propose a strategy which is inspired by other works on biological network analysis [18–20]. The basic idea is to define an objective function that leads to an adequate threshold after optimization. The objective function is based on quantitative graph properties (QGP). Three QGPs are selected and combined (see [21] for an overview) to determine *t*_S_. The total number of edges *N*_E_, the average clustering coefficient (*ζ*) [22] and the global efficiency (*ξ*) [23].

To calculate *t*_S_ we define a vector of candidate thresholds:

3 $$ \mathbf{t} = (t_{\text{min}}, \dots, t_{i-1}, t_{i}, \dots, t_{\text{max}}),  $$

where *t*_min_ and *t*_max_ are the minimum and maximum threshold, respectively and *t*_*Δ*_=*t*_*i*_−*t*_*i*−1_ is the step size to reach from *t*_min_ to *t*_max_. [*t*_min_,*t*_max_] defines the interval of threshold candidates in which we search for the best possible threshold to reduce the edges in our network. The interval is explored in a discrete manner. This implies that the resolution of the threshold detection is defined by *t*_*Δ*_, i.e. the distance between two consecutive points *t*_*i*_ to *t*_*i*+1_ in [*t*_min_,*t*_max_].

We calculate *N*_E_, *ζ* and *ξ* on each graph of an adjacency matrix *A*(*t*_*i*_) and arrange the results in vectors $\phantom {\dot {i}\!}\mathbf {\nu }^{N_{\mathrm {E}}}$, **ν**^*ζ*^ and **ν**^*ξ*^, respectively. Next, we use $\phantom {\dot {i}\!}\mathbf {\nu }^{N_{\mathrm {E}}} \mapsto [0,1]$ as baseline to adjust **ν**^*ζ*^ and **ν**^*ξ*^:

$$\begin{aligned} \mathbf{\eta}^{\zeta} &= \mathbf{\nu}^{\zeta} - \mathbf{\nu}^{N_{\mathrm{E}}} \\ \mathbf{\eta}^{\xi} &= \mathbf{\nu}^{\xi} - \mathbf{\nu}^{N_{\mathrm{E}}} \\ \end{aligned} $$

We create a mean centered matrix **X**=[**η**^*ζ*^,**η**^*ξ*^] and apply PCA as a weighting method. Therefore we calculate **y**, which is the projection of **X** on the first PCA component:

$$\begin{array}{*{20}l} \mathbf{X} &= \left[\mathbf{\eta}^{\zeta}, \mathbf{\eta}^{\xi}\right]& &\text{ and }& \mathbf{X}^{\text{cov}} &= cov(\mathbf{X}^{\mathrm{c}}) \\ \mathbf{X}^{\text{cov}} \mathbf{u}_{i} &= \lambda_{i} \mathbf{u}_{i}& &\text{ and }& \mathbf{y} &= \mathbf{X} \mathbf{u}_{0} \text{,} \end{array} $$

where **X**^c^ is the mean centered version of **X**, {**u**_*i*_} are the eigenvectors of the covariance matrix **X**^cov^ of **X**^c^ and *λ*_*i*_ are their respective eigenvalues labeled in decreasing order, *λ*_0_≥*λ*_1_≥…. To determine the final threshold we search for the candidate threshold for which the value of **y** is maximized. This leads to maximizing the weighted combination of the baselined average clustering coefficient *ζ* and the global efficiency *ξ*. Hence, we can set *t*_S_, with:

4 $$ \begin{aligned} \mathrm{S} = \arg \, \max_{k}\{\mathbf{y}_{k}\}\text{,} \, \, \,k = 0, 1, \dots, |\mathbf{y}| \}  \end{aligned}  $$

Since the primary objective is to achieve dense communities, it is a good choice to optimize a segregation measure like *ζ*. Nevertheless, we do not want to neglect the information provided from edges between communities and integrate *ξ*, which scales with integration. We use PCA as a weighting method because by construction *ζ* shows a higher variance than *ξ*. This leads to a stronger weighting. The idea to combine segregation and integration is based on the small-world property, which occurs frequently in biological networks [19]. The small-world property describes a graph structure of densely connected subgraphs that are interconnected by a robust amount of edges.

*N*_E_ serves as a baseline to avoid the effect that low thresholds produce high values for *ζ* and *ξ*, which is induced by the construction of these measures. This way the applied measures scale rather with structural properties than with the amount of edges. Since Pearson correlation (Eq. 2) serves as our similarity measure, we set:

*t*_min_=−1, *t*_max_=1, *Δ*=0.1.

For *t*_min_, *t*_max_, and *t*_*Δ*_ one has to balance computation time and resolution.

For considered alternatives we refer the interested reader to the Additional file 1: S17.

Now, **A** represents an undirected, unweighted graph **G**, which serves as basis for the community detection. In **G** each node *v*_*i*_, with *i*=1,…,*N*_V_, where *N*_V_=*#*nodes, corresponds to a single *m/z*-image and is called *m/z*-node, while each edge *e*_*k*_={*v*_*i*_,*v*_*j*_} indicates that: *w*_*ij*_>*t*_S_, with:

*k*=1,…,*N*_E_; *i*,*j*∈{1,…,*N*_V_} and *N*_E_=*#*edges.

For community detection we use the leading eigenvector method [8, 9]. This method proceeds in a divisive style and maximizes a measure called modularity [24]. Since this is a divisive method, for initialization each *m/z*-node *v*_*i*_ is assigned into the same community *c*, with:

*c*∈1,…,*N*_C_ and $v_{i}=v_{i}^{c=1} \ \forall \ i$,

where *N*_C_=*#*communities.

Thereafter, the method proceeds with:

1. For each existing community *c* its modularity matrix **M**^(*c*)^ is calculated. Informally speaking, for each pair of vertices (*v*_*i*_,*v*_*j*_) the respective modularity matrix entry $M_{i,j}^{(c)}$ shows the existing number of edges substracted by the expected number of edges between these vertices (for more detail see [8, 9]).
2. The leading eigenvector **u**^(*c*)^ of **M**^(*c*)^ is calculated, which is the eigenvector corresponding to the largest eigenvalue $\lambda _{\text {max}}^{(c)}$.
3.
  - If *λ*^(*c*)^>0: All $v_{i}^{(c)}$ are partitioned into two new communities by:
    $$v_{i}^{c} = \begin{cases} v_{i}^{(c)}, & \text{if}\ \mathbf{u}_{i} \geq 0 \\ v_{i}^{(c')}, & \text{otherwise} \end{cases} $$
  - else: label $v_{i}^{(c)}$ as “indivisible” and continue with a divisible community.

The procedure repeats for each community until all are labeled as “indivisible”. *λ*=0 is used as stop criteria as its **u**=(1,…,1), which means that the best division is to set all *v*_*i*_ in *c* and none in *c*^′^, i.e. the best division is no division.

It is important to mention that the original work [8, 9] does not explicitly mention how to handle disconnected components. However, for MSI data sets disconnected components can be assumed to be quite common. In order to deal with this problem we propose a slight modification of the algorithm, by changing the initialization. Instead of initializing every *m/z*-node in one community, we search for connected components and set each connected component in its own community. Using this as initialization we follow the leading eigenvector method as described above.

For alternative community detection methods we would like to refer the interested reader again to S17 of the Additional file 1. To facilitate the description of a community size we will use the terminology of (*n*)-Community, where *n* provides information about the size.

### Visualization

**Fig. 3:**
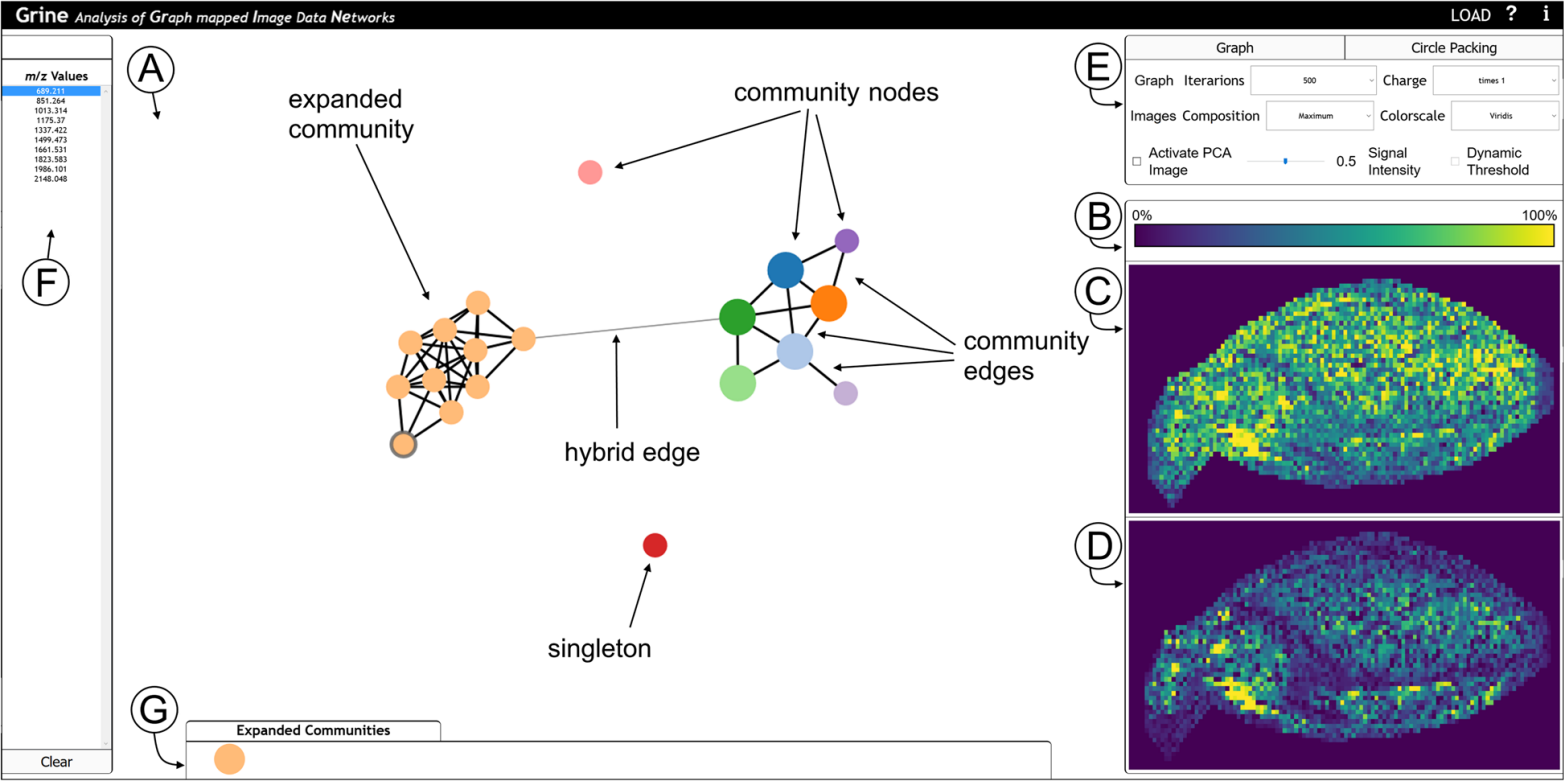
GRINE UI with graph mode active and hierarchy mode (circle packing) inactive. One community of the whole community-graph *G*^′^, which is shown in (**a**), is expanded and the *m/z*-node of *m/z*-value 689.211 is selected. (A) Network display in graph mode. (**b**-**d**) Image Display. **b** Legend for color scheme (in this case: viridis). **c** Community-map. **d***m/z*-image. **e** Options box to configure the graph, image and hierarchy mode. **f** List of all *m/z*-values or, if selected, of all *m/z*-values in the selected community. **g** Expanded communities

Molecular communities are characterized by two aspects that need to be explored simultaneously: localization and network structure. To analyse the computed communities in this regard, we propose an interactive visualization framework that links two visualizations for these two aspects. The tool is referred to as GRINE (Analysis of **GR**aph mapped **I**mage Data **NE**tworks) and can be tested for the data described in this paper using the provided links (availability or supplementary). The interface of the tool is shown in Fig. 3. The functionalities are motivated and described below.

To visualize and explore the **network structure display** the user can choose between two different modes: In **graph mode** the communities’ graph structures are visualized, starting with a community graph *G*^′^ (see Fig. 3a). Each community forms one node $v_{C_{i}} = \{v_{j}\}_{i}$, where {*v*_*j*_}_*i*_ is the set of all *m/z*-nodes with a community membership of *i*. Two community nodes are connected by a community edge $e^{\text {(C)}}_{k}$, with:

$\phantom {\dot {i}\!}e^{\text {(C)}}_{k} = \{v_{C_{i}},v_{C_{j}}\}$,

if there exists an edge *e*_*l*_={*v*_*p*_,*v*_*q*_}, with:

$\phantom {\dot {i}\!}v_{p} \in v_{C_{i}}$ and $v_{q} \in v_{C_{j}}\phantom {\dot {i}\!}$.

The graph is fully dragable and repositions itself by a force layout. The user has the option to expand a community to show its subgraph and edges $e^{\text {(H)}}_{k} = \{v_{C_{i}},v_{j}\}\phantom {\dot {i}\!}$ which we refer to as hybrid. Hybrid edges are edges between *m/z*-nodes and community nodes, meaning that an *m/z*-node of an expanded community is connected with an *m/z*-node of a non expanded community. Each node can be selected to activate the image display.

In **hierarchy mode** a circle packing is applied to visualize the networks while hiding the details of the graph structures (i.e. edges). This enables users to focus on community memberships instead (see the Additional file 1: S2 for a screenshot).

To analyse the **localization** of communities and community members, the user selects them either in the graph or in the hierarchy mode, which triggers the visualization of their spatial distribution in the **image display** (see Fig. 3c and d). The upper frame (Fig. 3c) shows the **community map** with a pseudo coloring chosen from a menu (Fig. 3e). The community map is a summary of all images from one selected community $I_{C_{i}} = D_{\mathbf {p},\{s_{j}\}_{i}}$, i.e. all *m/z*-images corresponding to *m/z*-values *s*_*j*_ that are members of community *C*_*i*_.

Community maps can be computed and visualized in two modes: In **maximum projection mode** the maximal intensity in the community is displayed for each pixel:

$$\Phi(p'_{k}) = \max_{s_{l}}(\Phi(p_{k,\{s_{l}\}_{i}}), $$

where *Φ*(*pk*′) is the intensity of pixel $p^{\prime }_{k}$. This mode displays the total area covered by the entire community.

In **averaging mode** the intensity for each pixel is averaged across all images in the community:

$$\Phi(p'_{k}) = \frac{1}{|\{s_{l}\}_{i}|} \sum_{l}\Phi(p_{k},\{s_{l}\}_{i}). $$

This emphasizes the quantity of signal coverage.

The lower frame (Fig. 3d) shows the single mass map visualizing one $I_{(m/z)_{i}}$ image (after selecting this community member in the network display or in the mass list on the far left (Fig. 3f)). The pixel intensities are rescaled for a maximum contrast to enable the visual analysis of weak mass signals.

Furthermore, there is the option to visualize the relation of community localizations with another kind of pseudocolor map, the PCA (principle component analysis) map. This visualization takes the full data set *D* into account and thus accounts for variances in the entire *N*_V_ dimensions. The R, G, B color values in the PCA map are computed with a projection of the full data set onto the three most informative principle components (details given in Additional file 1: S5). This map has been implemented to enable users to integrate global data features. In addition, PCA is a well established and familiar way to analyze high dimensional data so that it can be used as a reference despite its limitations.

Some implementation details can be found in S14 of the Additional file 1.

Finally, we would like to refer the reader to S16 of the Additional file 1 for further information on how the similarity measure, threshold selection and community detection algorithm influence each other and their impact on the downstream analysis.

## Results

Weblinks to all results obtained for data sets: *D*_G_, *D*_B_ and *D*_T_ can be found under **Availability of data and material**.

### Gaussians

**Fig. 4:**
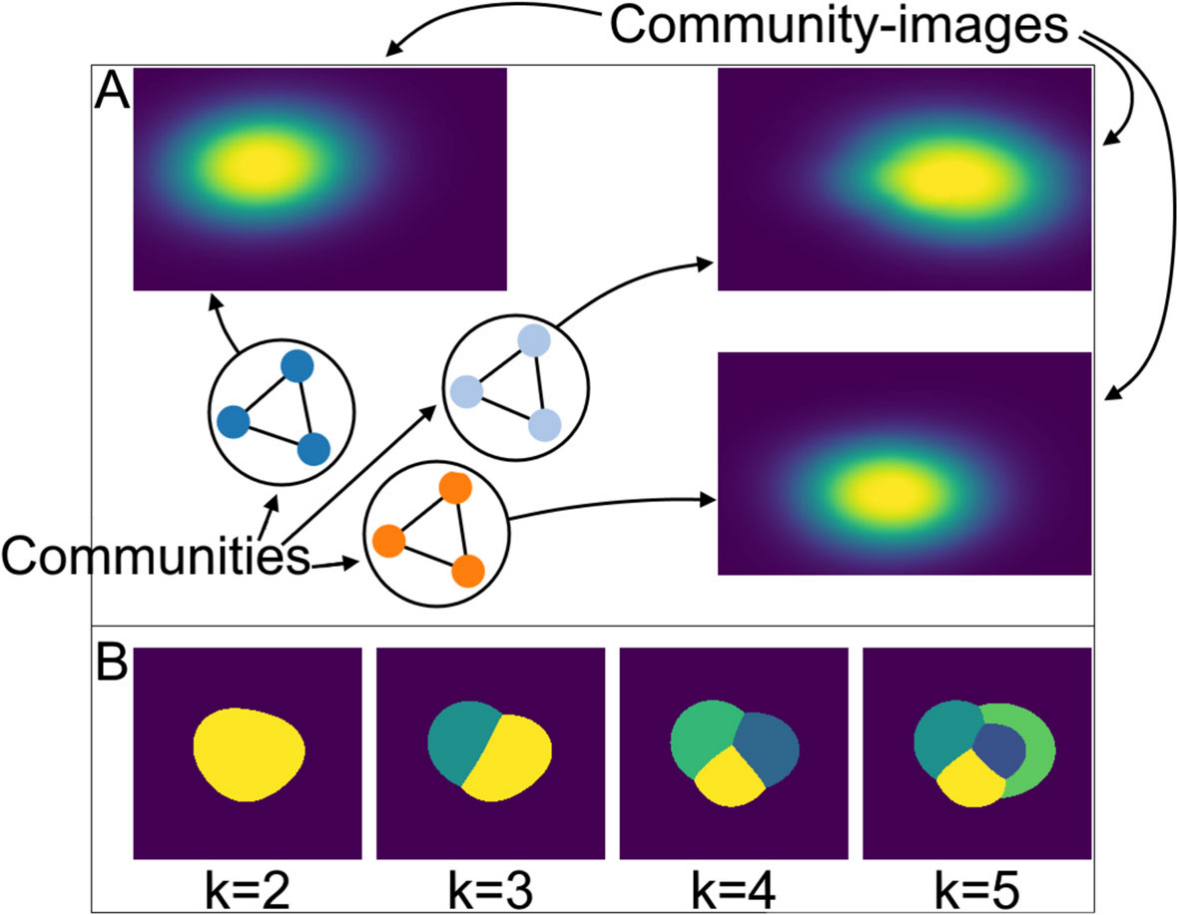
**a** Our proposed method was applied to the synthetical *D*_G_ data set. The three pseudo-networks were correctly detected as three communities. The communities are displayed as colored graphs (screenshot from the GRINE tool). For each community, the community-map is shown with a viridis color map. **b***k*–means segmentation map after clustering of pixel, i.e. *m/z*-spectra, for *k*=2,…,6. Each color represents one cluster

For the data set *D*_G_ an edge reduction threshold within *t*_S_∈[0.6382,0.9397] was computed (see Table 1 and Eq. 4). The specific value picked inside of this interval is irrelevant, since the arg max function is maximal over the entire interval. Our community approach detects three communities that corresponds to the groups $K^{\text {gs}}_{i}$, with *i*=0,1,2, meaning that we can distinguish the gaussians based on their spatial location (see Fig. 4a).

**Table 1:** Summarized graph information

Dataset	*t*_S_	*N*_E_	*N*_V_	*N*_C_	$\phantom {\dot {i}\!}N_{\mathrm {C}_{2+}}$
Gaussian Circles (*D*_G_)	0.6382	9	9	3	3
Barley (*D*_B_)	0.7085	789	101	11	8
Glioblastoma (*D*_T_)	0.5477	2371	106	11	6

Threshold for edge reduction (*t*_S_), number of edges (*N*_E_), number of vertices (*N*_V_), number of communities (*N*_C_) and number of communities of size greater than two ($\phantom {\dot {i}\!}N_{\mathrm {C}_{2+}}$) for *D*_G_, *D*_B_ and *D*_T_ are shown

If we discuss this result in relation to our biological analogy, each group $K^{\text {gs}}_{i}$ with distribution at $L^{\text {gs}}_{i}$ consists of molecules that are likely to be representatives of a metabolic process located in this area. Let us remember our initial assumption that functionally related molecules feature a similar lateral distribution within the sample, i.e. metabolic processes are spatially bound. If this assumption holds, the results obtained from *D*_*G*_ indicate that our communities can help to: 1. distinguish metabolic processes based on their spatial location and 2. identify their important molecules.

Figure 4b shows *k*-means segmentation maps with different *k*, i.e. clustering of pixel. Even with the correct number of clusters (*k*=4, i.e. background and three pseudo-networks) the segmentation map cannot distinguish the covered areas at the three different locations.

Compared to *k*-means clustering or hierarchical clustering, our method does not require to determine the number of groups, which can be considered an advantage.

### Barley

For data set *D*_B_ we computed the threshold *t*_S_=0.7085 (Eq. 4). This results in *N*_E_=789 edges, meaning a reduction of 84.376*%* (Table 1). Based on the resulting graph, the leading eigenvector method found *N*_C_=11 communities (see Additional file 1: S4). Nine of them are interconnected, while two are singletons, i.e. nodes without any edge. Eight of the interconnected communities are (*n*)-Communities, with *n*>1, the others are (1)-Communities.

**Fig. 5:**
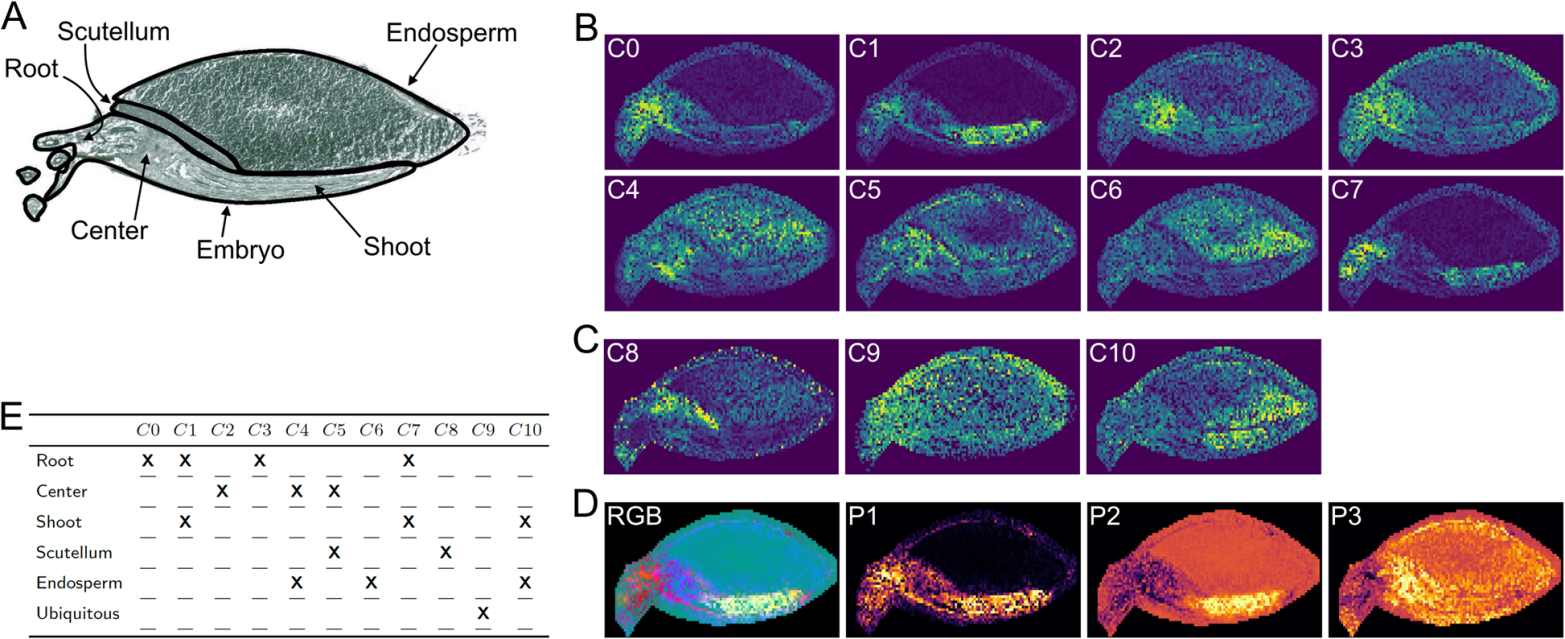
**a** Optical image scan with marked and labeled anatomical structures. **b** Average community-maps of all (*n*)–communities, with *n*>1 (network in Additional file 1: S4). **c** Images of (1)–Communities (network in Additional file 1: S4). **d** RGB image of the first three PCA projections, where the projections on the eigenvectors of the first, second and third largest eigenvalue is assigned to the red, green and blue channel, respectively and standalone images of these components. PCA images are not scaled like the community-maps and *m/z*-images. The color map viridis is used for images in (**b**) and (**c**) and inferno for images in (**d**). **e** Correlation between the spatial signal distributions of all found communities and the anatomical structures of the barley seed. **X** indicates that a community shows increased signal in the respective area

Most signal distributions of the community maps (Fig. 5) show a strong correlation to anatomical structures of the barley seed, which is summarized in Fig. 5e.

**Fig. 6:**
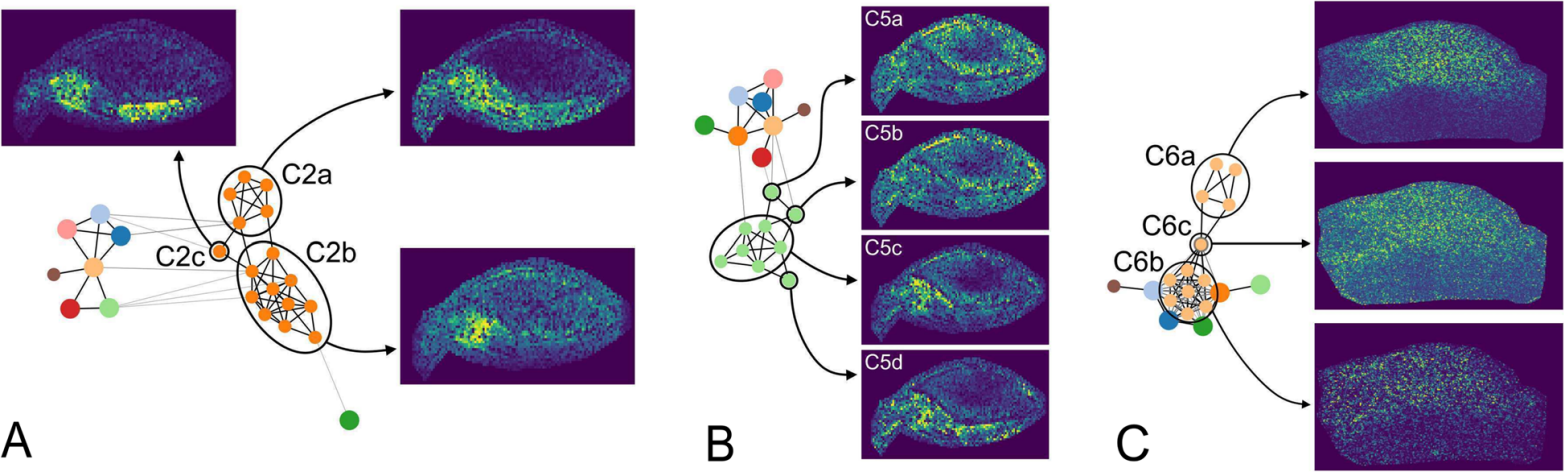
**a** Substructures of *D*_*B*_ in community *C*2. The whole graph of *D*_B_ is shown with the corresponding community-node *C*2 unfolded. The substructures are encircled and refer to their respective subcommunity-maps. **b** Core-offshoot structure of *D*_*B*_ in community *C*5. The left side shows the graph of *D*_B_ with *C*5 unfolded. The core structure and the offshoots are encircled. The right side shows the core-community-map and the *m/z*-images of the offshoots. **c** Substructure of *D*_*T*_ in community *C*6. The left side shows the graph of *D*_T_ with *C*6 unfolded. The two substructures, as well as their connecting link (single node), are encircled. The right side shows the subcommunity-maps of the marked nodes. For all images the color map viridis is used

A view on the graph structure of *C*2 (Fig. 6a) reveals that this community can be divided into more detailed sub-communities (referred to as *C*2*a* - *C*2*c*). *C*2*b* shows an increased signal only at the embryo center, while the signal of *C*2*a* is less specifically distributed in the entire embryo. *C*2*c* is located between both and shows a specific signal distribution at the center and the shoot. A similar observation can be found for *C*5. The subgraph of *C*5 (Fig. 6b) shows a structure that can be distinguished into core and offshoots. A core is defined by nodes that are densely interconnected, while offshoots are reaching out from the core and are less interconnected. The core of *C*5 (*C*5*c*) defines the main signal distribution of this community, which extends from the scutellum into the embryo center. The three offshoots *C*5*a*, *C*5*b*, and *C*5*d* deviate from this distribution. A similar core-offshoot differentiation can be observed in *C*4 (not shown).

The identification of *m/z*-values based on prior examination of barley seed MSI [12] reveals a tendency for communities to mostly contain one class of molecules. *C*0, *C*1, *C*3 and *C*7 contain only hordatines and hordatine precursors, with one exception in *C*0, which is a lipid and three exceptions in *C*3, which are two unknown molecules and one lipid. *C*2 and *C*4 contain mostly carbohydrates, with four exceptions (three unknown molecules and one lipid). Further, carbohydrates in *C*2 are only potassium adducts and in *C*4 only sodium adducts. *C*5 and *C*6 contain mostly lipids, with two exceptions in *C*5 that are unknown molecules. The (1)-Communities are unknown (*C*8, *C*9) and a lipid (*C*10). This indicates that similar molecules have similar spatial distributions. One reason for this could be that similar molecules are part of the same spatially bound metabolic processes.

The identification also supports the structural features of *C*2 and *C*5. *C*2*a* is composed of three unknown molecules, one lipid and one carbohydrate, while *C*2*b* consists only of carbohydrates. For *C*5, the two images that fit least to the main signal distribution of the community are both unknown molecules.

### Glioblastoma

For data set *D*_T_ we computed the threshold *t*_S_=0.5477 (Eq. 4). The result is *N*_E_=2371 edges, i.e. a reduction of 57.394*%* (Table 1). Compared to the barley data set the number of edges is clearly higher, although the number of vertices is nearly equal. The reason is a higher general similarity and a lower spread of similarity values, i.e. the algorithm classifies more similarities to be relevant. This indicates a higher degree of complexity for the tissue and its respective network of functionally related molecules. The community detection result shows *N*_C_=11 communities with seven of them interconnected (see Additional file 1: S4). Five are (1)-Communities, the other six are (*n*)-Communities, with *n*>1.

**Fig. 7:**
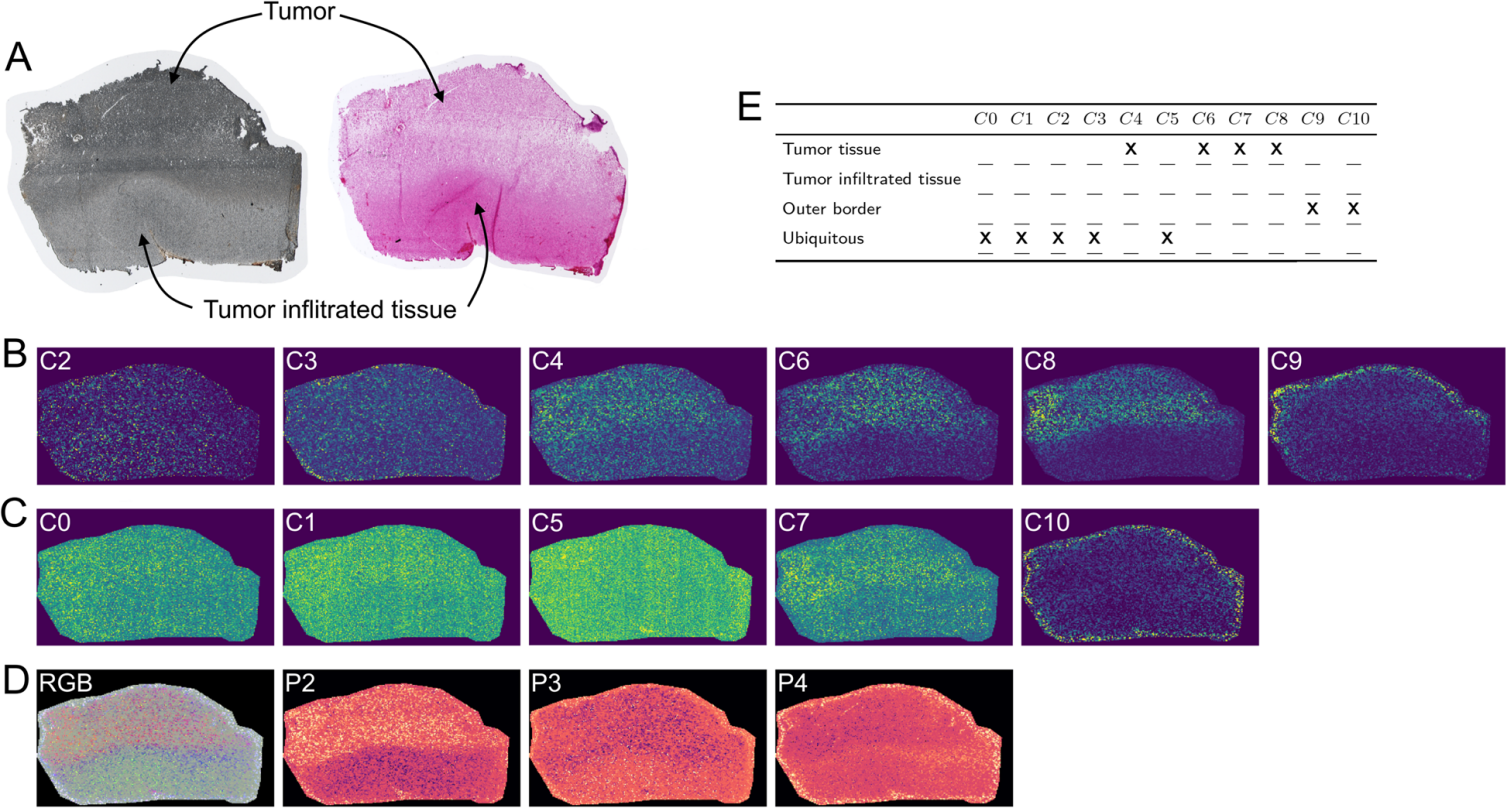
**a** Optical image scan of the sample used for MALDI analysis (left) and H&E stained image scan of the subsequent sample section (right). For the H&E stained image lighter color indicates tumor tissue and darker color indicates tumor infiltrated tissue, while this is reversed for the optical image. **b** Average community-maps of all (*n*)–Communities, with *n*>1 (network in Additional file 1: S4). **c** Images of (1)–Communities (network in Additional file 1: S4). **d** RGB image of the second, third and fourth PCA components, where the projections on the eigenvectors of the second, third and fourth largest eigenvalue is assigned to the red, green and blue channel, respectively and standalone images of these components. PCA was done without the additional preprocessing steps of data squaring and image thresholding. The PCA images are not scaled like the community-maps and *m/z*-images. The color map viridis is used for images in (**b**) and (**c**) and magma for images in (**d**). **e** Allocation of the spatial signal distribution of all found communities to specific pattern within the glioblastoma sample. We determine three main areas: Tumor tissue, tumor infiltrated tissue and outer border. **X** indicates that a community shows increased signal in the respective area

The signal distributions (Fig. 7) reveal three main patterns, which are summarized in Fig. 7e.

Similar to the results obtained for barley data, a detailed view on the graph structure reveals more detailed information (Fig. 6c). The subcommunity *C*6*a* shows a strong and specific distribution in one half of the sample. *C*6*b* is distributed notably less specific, with a slightly biased signal distribution to the same half of the sample as *C*6*a*. Both subcommunities are connected by a *m/z*-image (*C*6*c*) that shows a weak similarity to *C*6*a*. We assumed that *C*6*c* produces a chaining effect during the community detection.

Based on communities *C*6*a* and *C*8 we can conclude that the sample is functionally divided into two halves, which is in line with the PCA result (Fig. 7d) and (more important) the H&E staining information (Fig. 7a), which indicates that the tumor in this sample is side specific. We can presume that at least some molecules of *C*6*a* and *C*8 could be tumor specific.

Results of the publicly available mouse urinary bladder data set from ms-imaging.org are shown in Additional file 1: S12. There we provide some basic results without detailed biological interpretation. The results are available for exploration in our webtool. The respective link can be found in Additional file 1: S1.

## Discussion

### Barley

The analysis of the barley seed data set shows that the community analysis approach delivers reasonable results, i.e. the spatial localizations of the communities reflect biological compartments with distinct functions. This is in accordance with previous findings for this data set [12]. For most communities, we are able to clearly detect correlations with different anatomical structures.

In contrast to other established methods for MSI segmentation, the presented approach offers a very fine identification of the different tissues of a barley seedling based on the mass spectroscopy data. As shown in Fig. 5, the root, the center of the developing seedling, the shoot, the scutellum, and the endosperm could be identified by a unique combination of communities. This segmentation can be used to analyze the co-localization of specific single mass channels, representing known intermediates of the metabolism.

The fact that certain tissue regions or organs are represented by a number of different communities indicates that these parts of the sample are physiologically more heterogeneous than would be expected if a single *m/z*-signal were co-localized with that particular tissue or organ. An example for this kind of heterogeneity for the shoot can be seen in the communities *C*1, *C*7, and *C*10. Most interestingly, it shares communities with the root, but not with the scutellum. From a biological point of view, it can be speculated that these differences reflect metabolite compositions that are characteristic for developing tissues, as roots and shoots, versus a tissue, which is metabolically active but not further developing just like the scutellum.

The appearance of substructures in individual communities within the graphs illustrates that our graph approach is able to convey information that would remain hidden if just cluster results were considered. Interestingly, the three substructures investigated in this study show already three different kinds of motifs: Simple subgroups, core-offshoot structures, and bridging (or chaining) structures. Therefore we believe that substructures are worth further examination.

### Glioblastoma

The results of the glioblastoma data set are not as easy to interpret as those of the barley sample, which was to be expected. This is due to its morphological homogeneity, combined with heterogeneity of the cell phenotype. On the other hand the community detection yields at least one clear insight: There are groups of molecules, whose signal distribution correlate with the tumor area that was defined by a pathologist [13]. This provides candidates for subsequent biological experiments.

Regarding their community compositions, the tissue compartments classified as tumor and tumor-infiltrated in data set *D*_*T*_ are much more similar to each other than the different compartments of the barley sample. Five of the eleven communities are categorized as ubiquitous (Fig. 7), reflecting the fact that the tumor tissue is still closely related to the non-tumor tissue. Four communities are tumor-specific (Fig. 7), probably induced by the localization of lactate and other tumor metabolites (see [13]). The last two communities refer to the outer border of the sample (Fig. 7), probably induced by matrix peaks.

We believe that even without any prior knowledge about the sample, like H&E staining, the results offered by this type of analysis provide a good starting point for biologists to set up further experiments.

### Visualization

Our visualization tool GRINE is interactive, dynamic and responsive. This makes the usage very intuitive and almost no learning phase is required. The tool shows its main strengths in three areas. First, it combines the information of the graph domain and the image domain. Second, the interaction with the graph facilitates the focus on specific communities and allows to spot structural characteristics. Examples are: Substructures that can indicate more finely resolved communities, cluster ambiguities and potential misclusterings. Third, its possibility to show and hide information, i.e. its interactivity, allows to encode much more information in a clear way than we could achieve with static visualizations [25], e.g. average and maximum images of all communities and correlation with PCA results.

At the current time, the visualization can only deal with distinct communities, whereas the analysis pipeline can also search for overlapping ones.

### Comparison to other methodological approaches

A more common approach than the one presented for the analysis of the spatial distribution of imaging data is to employ dimension reduction techniques for segmentation. We compared our method to visualizations of three different dimension reduction techniques: principal component analysis (PCA), non-negative matrix factorization (NMF) and latent dirichlet allocation (LDA) (results are shown and discussed in Additional file 1: S13). We decided for PCA as it is probably the most prominent dimension reduction technique in biology. NMF is also a commonly used technique and does not produce negative intensity values, which can occur in PCA. LDA was chosen because it is a generalization of pLSA (probabilistic latent semantic analysis) that has been previously analysed [7].

The comparison showed that the computed visualizations reveal similar coarse grained structures as our method. It is worth noting that LDA performs better as NMF and NMF performs better than PCA. For *D*_*B*_ and *D*_*T*_ the segmentation maps of LDA reveal the most details and detected structures show the highest contrast. This is followed by the ones obtained with NMF. The PCA maps provide the lowest contrast. All three methods show distributions that correlate with the main structures of the samples. However, compared to our method they fail finding finely detailed structures like the scutellum in *D*_*B*_.

While the results obtained with PCA, NMF and LDA share similarities with the results obtained by our proposed method, we can report some new favorable features for our approach:

First, the grouping of spatial distributions assigns each image to one group. After analysing the lateral distribution of a community image it is easy and unambiguous to identify which single *m/z*-images, i.e. molecules, participate in this distribution. This is much harder for PCA, NMF and LDA, where each component image consist of partial combinations of the original *m/z*-images.

Second, we do not need to determine the number of clusters, i.e. communities, beforehand. Our method chooses this number automatically based on the given optimization criterion (modularity). If needed, a manual decision is still possible. This is different for NMF and LDA. For those methods the number of dimensions, i.e. components, have to be predefined. Finding the most fitting number of dimensions for a given sample is a non trivial task and especially important for NMF and LDA, since the number of dimensions influences the lateral distribution of the resulting components (see Additional file 1: S13).

Third, the community images are based on simple aggregation functions. Therefore, in case of outliers or ambiguities it is easy to re-evaluate the community images without them. The same counts for potential optimizations based on substructures in the clustering space.

Fourth, the network structure can reveal outliers, misclusterings, substructures and potential optimizations at first glance and allows an intuitive exploration of the clustering space.

We would also like to add the H^2^SOM ([5]), as another segmentation method to this comparison discussion. This method is also capable to reveal detailed structures in *D*_*B*_. A core difference is that in our method a single pixel can be a member of multiple community images. This means we can provide an ambiguous pixel labeling, which is more suitable to represent dynamic biological processes.

## Conclusion

In this paper we demonstrated the general applicability of community detection as an unsupervised clustering technique for the analysis of MSI data from different types of samples. We have developed a pipeline to map lateral image data to an image similarity graph. We have also developed a new edge thresholding technique to transform a fully connected graph into a sparse one. Using lateral *m/z*-images as samples and their pixels as features, we utilized community detection as an example to group molecules with similar lateral distributions. By analysing the network structure with our interactive visualization, we have found finer subclusters within the detected clusters. This offers a possibility for manual refinement.

We stated the initial assumption that functionally related molecules are spatially bound. If this assumption holds, the presented way of clustering lateral imaging data provides a good starting point for targeted biological experiments. For some information on the limitations of this assumption and alternative considerations, we would like to refer to S18 of the Additional file 1.

This paper is designed as proof of concept to demonstrate the general applicability of community detection to MSI data. Therefore, we did not discuss the question of performance. Since we do not want to ignore this question entirely, we refer to S15 of the Additional file 1. There a rough analysis of the complexity is presented.

Finally, a webtool has been implemented to visualize and explain the results and to demonstrate the usefulness and benefits of the approach.

***Future research*** Further analysis of network patterns of substructures could lead to automated ways to detect finer cluster relationships, like hierarchical structures and reveal and correct misclusterings. Another promising approach, which was not discussed in this paper, is to employ more statistical network properties for the analysis. An example could be the ratio of the in-group degree to the out-group degree to automatically detect very specific or very general lateral patterns. These statistics could also be used to query specific *m/z*-images for their statistical properties. Considering the sheer amount of network properties this offers a big area for future research.

## Additional file

Additional file 1 Supplementary. (PDF 2459 kb)

## Abbreviations

H ^**2**^SOM: Hierarchical hyperbolic self-organizing map
H&E: Hematoxylin and eosin
GRINE: Analysis of **GR**aph mapped **I**mage data **NE**tworks
LDA: Latent dirichlet allocation
*m/z*: Mass to Charge
MALDI: Matrix-assisted laser desorption ionization
MSI: Mass spectrometry imaging
NMF: Non-negative matrix factorization
PCA: Principal component analysis
pLSA: Probabilistic latent semantic analysis
QGP: Quantitative graph properties
