# Supplementary material for: Detection and visualization of communities in mass spectrometry imaging data
Source: BMC Bioinformatics. 2019 Jun 4;20:303. doi: 10.1186/s12859-019-2890-6 (PMC6549267; doi:10.1186/s12859-019-2890-6)
Supplement: Supplementary file 1 — Supplementary. (PDF 2459 kb) [file 12859_2019_2890_MOESM1_ESM.pdf]

Supplementary

S1. Availability

Gaussian result:
https://kawue.github.io/grine_demo/index_gaussians_githubpages.html

Barley result:
https://kawue.github.io/grine_demo/index_barley_githubpages.html

Glioblastoma result:
https://kawue.github.io/grine_demo/index_glioblastoma_githubpages.html

Mouse urinary bladder result: https://kawue.github.io/grine_demo/index_bladder_githubpages.html

Network building and community detection code:
https://github.com/Kawue/imaging_communitydetection_demo

Visualization (Grine) code:
https://github.com/Kawue/grine_demo

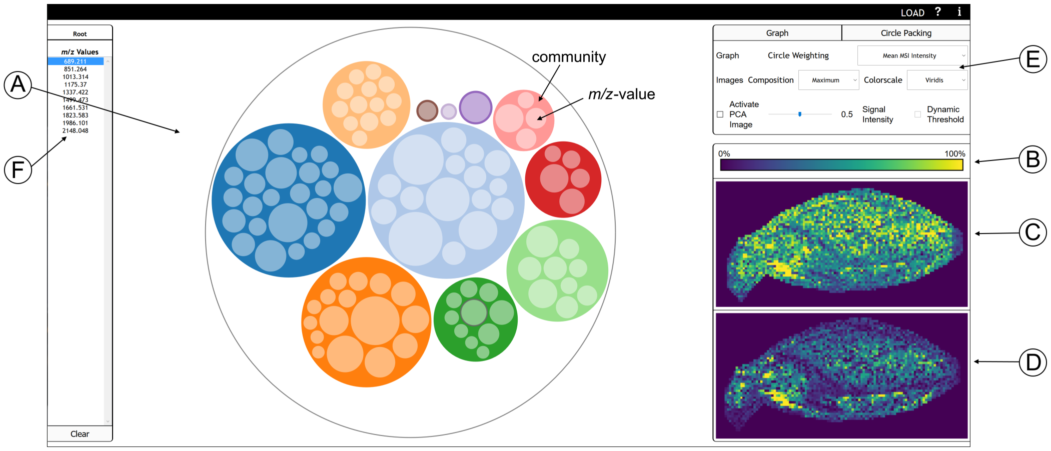

Figure 12: Grine UI with network hierarchy mode active and graph mode inactive. The circle of m/z-value 689.211 is selected. (A) Network display in hierarchy mode. (B-D) Image Display. (B) Legend for color scheme (in this case: viridis). (C) Community-map. (D) m/z-image. (E) Options box to configure the graph, image and hierarchy mode. (F) List of all m/z-values or, if selected, of all m/z-values in the selected community.

S2. Circle packing mode in network display

Figure 12 shows an example of the hierarchy mode (circle packing).

S3. Calculation of two dimensional gaussians

The gaussians were calculated by:

$$O^{\text{gs}} = Ae^{-(a(x-x^{\text{c}})^2+2b(x-x^{\text{c}})(y-y^{\text{c}})+c(y-y^{\text{c}})^2)}$$

$$a = \frac{\cos^2(\Theta)}{2\sigma_x^2} + \frac{\sin^2(\Theta)}{2\sigma_y^2},\ b = -\frac{\sin(2\Theta)}{4\sigma_x^2} + \frac{\sin(2\Theta)}{4\sigma_y^2}$$

$$c = \frac{\sin^2(\Theta)}{2\sigma_x^2} + \frac{\cos^2(\Theta)}{2\sigma_y^2},$$

where $A \in [5, 6)$ is the amplitude, $(x^{\text{c}}, y^{\text{c}})$ is the center of the gaussians, σ_x and σ_y is the spread in x and y direction and Θ is the rotation angle. Randomized distortion of $x^{\text{c}}, y^{\text{c}}, \sigma_x, \sigma_y$ is achieved by adding ϵ to each value, where ϵ is uniformly sampled on the interval $[-5, 5)$.

S4. Graph Structures of Barley and Glioblastoma

Figure 13 and Figure 14 show the calculated community network structure for data set D_B and D_T, respectively. Both networks can be interactively explored

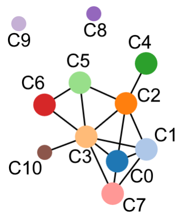

Figure 13: Community-graph of the barley seed sample. Each node symbolizes a community. Each community has a different color assigned. Smaller vertices symbolize (1)–Communities.

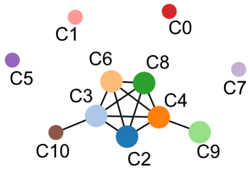

Figure 14: Community-graph of D_{T}. Each node symbolizes a community. Each community has a different color assigned. Smaller nodes symbolize (1)–Communities.

by visiting the respective links given under **Availability of data and material**.

S5. Correlation between community-maps / m/z-images and PCA

A PCA component image I^{PCA} is computed and used as background canvas. Data projections on the three most informative eigenvectors (PCA components) are assigned to the red, green and blue color channel, respectively, like in Fonville *et al.* (2013). These eigenvectors mostly correspond to the three largest or the second to fourth largest eigenvalues. If this option is selected the image display changes. If no community-map is displayed, the full PCA component image is shown. If a community-map or an m/z-image is displayed depending on the chosen mode (threshold based or dynamic) their presentation change. In **threshold mode** pixels above a chosen threshold are transparent, showing the I^{PCA} background, all others are greyed out. In **dynamic mode** the transparency scales with the signal intensity. Both modes are demonstrated in Figure 15. There are multiple reasons to include the PCA result in our visualization. First of all, PCA is one of the most established data analysis methods and most biologists have experience in interpretation of PCA results. As our community results are likely to be new for biologists, comparison to results of a known method, like PCA, can support the interpretation of communities. Second, PCA often succeeds in showing the main signal distributions of the underlying data set.

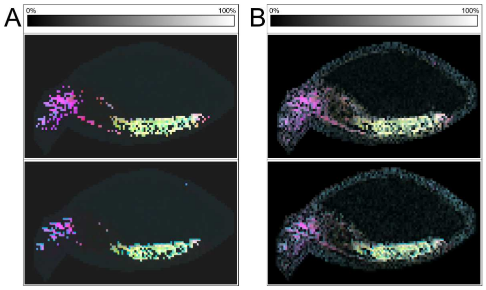

Figure 15: Correlation of a community-map and an m/z-image with the PCA component image. Signal intensity threshold is set to (A) 0.5, meaning every signal below 50% of the maximal signal is greyed out, and (B) dynamic.

S6. Comparison communities and PCA – Barley

The first three PCA components, ordered by decreasing eigenvalue, reveal five major areas that match the anatomical structure (Figure 5 D). The first component divides the seed in embryo and endosperm. The second component separates the shoot from the remaining embryo and the third component separates root and center from each other. Thus, these results are similar to our community approach, although it is coarser. Fine structures, like the scutellum or structural distinction within the endosperm are not detected. Additionally the second and third PCA components are visually not as easy to interpret as our communities, as they are not just a differentiation in signal and no signal. Moreover, to assign specific m/z-values to detected pattern, the PCA loadings have to be analyzed, as they show the importance of each m/z-value for each component. This is more complicated and more fuzzy than our method, or discrete clustering in general, where clear groups of m/z-values for each detected signal distribution are provided.

On the other hand, instead of just comparing our results with PCA we can use it as additional source of information and as a tool for first quality control like shown in Figure 15. If the signal distribution of a community overlaps strongly with the distribution of a PCA component or forms a subset of it, this can be seen as first evidence for a biological foundation instead of an methodologically conditioned artificial result.

S7. Comparison communities and PCA – Glioblastoma

PCA is calculated without the additional preprocessing steps of data squaring and image thresholding. Its evaluation shows, that the first component is not as rich in information, as the second, third and fourth, ordered by decreasing variance. Therefore we selected these three for analysis. Figure 9 D shows three major signal distributions. Signals in $P2$ and $P3$ are one sided distributed opposite to each other. $P4$ shows a weakly increased signal at the border of tumor and non tumor area, as well as at the outer border of the sample. The

split in two major areas is in line with both, our community results and the HE staining. Special to this result is the explicit revelation of a border area.

S8. Noise reduction

The glioblastoma data set D_{T} features more noise than the barley data set D_{B}. Therefore, we applied two additional preprocessing steps for D_{T}. First all intensities are squared: $D_{\mathrm{T}} = (D_{\mathrm{T}})^2$ to broaden the range of values, which already successfully suppresses a lot of noise. Second, image thresholding based on *Lis Minimum Cross Entropy method* Li and Lee (1993); Li and Tam (1998) is applied:

$$(D_{\mathrm{T}})_{p_k,s_l} = \begin{cases} 0, & \text{if } (D_{\mathrm{T}})_{p_k,s_l} < t_{\mathbf{p},s_l} \\ (D_{\mathrm{T}})_{p_k,s_l}, & \text{otherwise} \end{cases}$$

where $(D_{\mathrm{T}})_{p_k,s_l}$ is the k-th pixel of the m/z-image corresponding to m/z-value l and $t_{\mathbf{p},s_l}$ is Li's threshold calculated across all pixels of the m/z-image of m/z-value l. Li's method was chosen, because it does not need any parameter and denoises the image while maintaining main structural features.

S9. Comparison community detection, k-means and hierarchical clustering

A comparison of our community detection approach with k-means and hierarchical clustering is shown in Figure 16. Although all three methods are capable of finding the correct groups, k–means and hierarchical clustering need the correct number of groups as prior knowledge, while our community detection approach does not. Knowledge about the number of groups is often unavailable, giving our approach an advantage over methods that require it.

S10. Image scaling

As explained in the methods part, we decided to scale each image separately based on their intensity wise weakest and strongest pixel, before using them in Grine to improve the perception of detected pattern:

$$\begin{aligned} \Omega_{\min} &= \min_k \Phi(p_k) \\ \Omega_{\max} &= \max_k \Phi(p_k) \\ \Phi(p_k) &= \Omega(p_k), \end{aligned} \tag{1}$$

where Ω is a function that maps the intensity values to a new domain according to the used continuous color scale.

There are two obvious alternatives to this scaling. Either to scale each image based on the weakest and strongest pixel across *all* images or to scale community wise and use the weakest and strongest pixel across all images in the same community. The advantage of scaling over all images is that background signals are reduced. However, a strong disadvantage is that images with

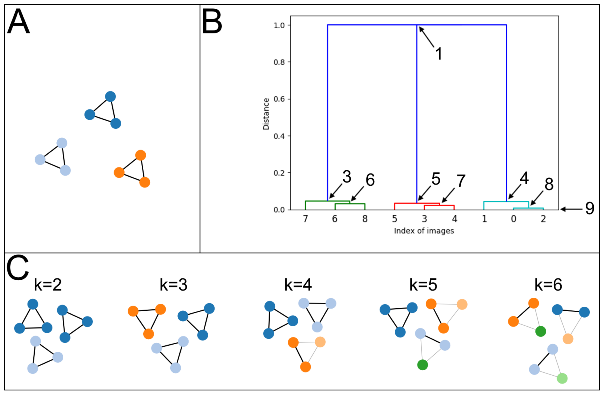

Figure 16: Comparison of three cluster methods: (A) Community detection with Grine. (B) Hierarchical clustering, based on average linkage and correlation distance. Numbers give the number of clusters if the hierarchy is split after the joint indicated by the arrow. (C) k–means for $k = 2, \ldots, 6$, based on the full similarity matrix. Each node is a $2D$ - gaussian image and each edge is the similarity between two connected nodes, assumed it is above $t^{\mathrm{S}} = 0.6382$. Different colors of nodes indicate different community membership.

very intensive signals can dominate the community-maps, weaker signals can be overshadowed and images with weak overall signal are hard to perceive or the signals are not perceivable at all. The advantage of scaling each image independently (Equation 1) is that all images contribute with the same strength to the community-maps and their signal is always perceivable. The main disadvantage is that background signals become stronger. Scaling per community behaves somewhere in between. The concrete behavior differs for each community and depends on their image composition.

In agreement with three of the authors (MG, HB, KN) we decided that the equal contribution of weak signal distributions in the community-maps is more important than reduced noise. Especially because, although the noise is stronger, localized spatial distributions can still be distinguished from background. It is important to mention that in this work these scalings only influence the visual perception and do not affect the similarity calculation. The reason is that the Pearson correlation coefficient is invariant to scaling. However, while using a similarity measure which is not invariant to scaling one have to scale the data before calculation. Furthermore, the influence of this scaling must then be kept in mind while analyzing the results. For such similarity measures, community wise scaling is obviously disqualified, since the scaling happens before the communities are detected.

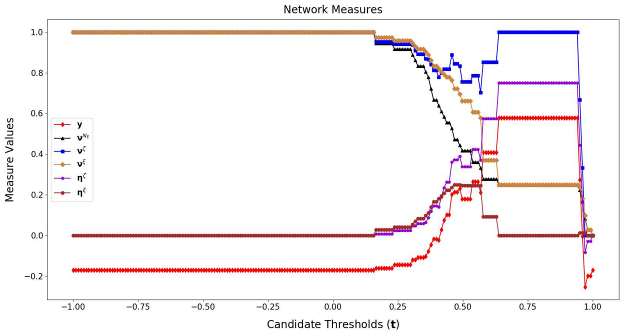

Figure 17: Line chart of the calculation of the final threshold t_S. Each line represents the vector of a relevant measure for the threshold calculation procedure. x-axis represents the candidate thresholds and y-axis the calculated value of the respective measure at a specific candidate threshold. The measure vectors are: Eigenvector projection ($\mathbf{y}$), total number of edges (ν^{N_E}), average clustering coefficient(ν^{ζ}), global efficiency (ν^{ξ}), baselined average clustering coefficient (η^{ζ}), baselined global efficiency (η^{ξ}). t_S is chosen as the maximum of $\mathbf{y}$

S11. Threshold selection

17, 18, 19 show the results of the threshold analysis of the data sets D_G, D_B, D_T, respectively. The final threshold t_S is selected at the maximum value of the projection on the first eigenvector y.

S12. Mouse Urinary Bladder

Here we present the results of a mouse urinary bladder data set, to show that our presented method is applicable to data sets that were not produced by us. This data set was downloaded from the website: *ms-imaging.org*. It is a publicly available and commonly known example data set.

The data was rebinned into 20000 bins by in house software. Based on the average spectrum the software mMass (see Strohalm *et al.* (2008, 2010); Niedermeyer and Strohalm (2012)) was used for peak picking with default options. The resulting peak list was used to pick the 150 most intense peaks.

The community detection results in 23 communities, with 17 of them are (1)-Communities (Figure 20). Most of these (1)-Communities are completely black. This is due to the winsorizing preprocessing step. Originally those images had irregularly distributed one pixel intensity spikes. Since those are assumed to be artifacts they are removed, leaving a black image. In the following we consider only (n)-Communities. Four main structures are revealed. Figure 21 (B) (C) show the innermost structure. Figure 21 (F) has most of its intensity distributed right around the innermost structure. Figure 21(G) has most of its intensity within the tissue but outside of Figure 21 (F) and Figure 21 (D) has most of its signal outside of the tissue, while Figure 21 (E) is complementary to

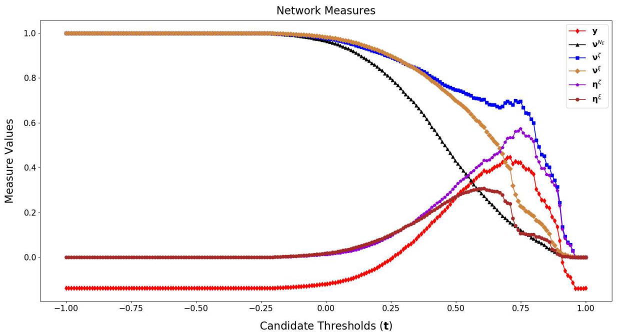

Figure 18: Line chart of the calculation of the final threshold t_S. Each line represents the vector of a relevant measure for the threshold calculation procedure. x-axis represents the candidate thresholds and y-axis the calculated value of the respective measure at a specific candidate threshold. The measure vectors are: Eigenvector projection ($\mathbf{y}$), total number of edges (ν^{N_E}), average clustering coefficient(ν^{ζ}), global efficiency (ν^{ξ}), baselined average clustering coefficient (η^{ζ}), baselined global efficiency (η^{ξ}). t_S is chosen as the maximum of $\mathbf{y}$

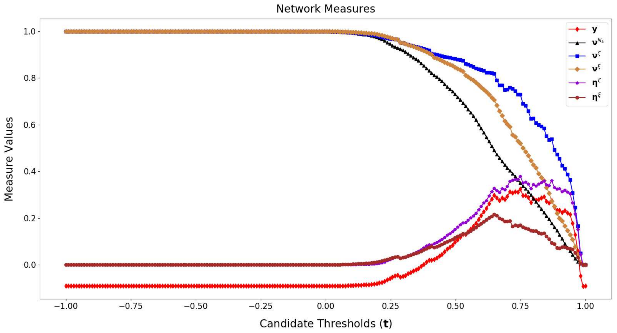

Figure 19: Line chart of the calculation of the final threshold t_S. Each line represents the vector of a relevant measure for the threshold calculation procedure. x-axis represents the candidate thresholds and y-axis the calculated value of the respective measure at a specific candidate threshold. The measure vectors are: Eigenvector projection ($\mathbf{y}$), total number of edges (ν^{N_E}), average clustering coefficient(ν^{ζ}), global efficiency (ν^{ξ}), baselined average clustering coefficient (η^{ζ}), baselined global efficiency (η^{ξ}). t_S is chosen as the maximum of $\mathbf{y}$

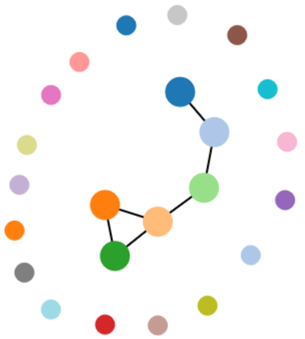

Figure 20: Community-graph of the mouse urinary bladder sample. Each node symbolizes a community. Each community has a different color assigned. Smaller vertices symbolize (1)–Communities.

Figure 21 (D). There is also a more specific distinction between Figure 21 (B) and Figure 21 (C). While (B) shows a strong one sided intesity bias, this is not the case for (C).

A detailed look at the subgraph of one of the communities (Figure 22) reveals that the whole community, except one m/z-node, is densely connected (Figure 22 (E)). This node is the only one which shows a stronger association with the patter of the orange community (Figure 21 (G)) instead of the actual assigned green community (Figure 21 (F)). Tracking the edges of this node shows that it is connected to three other nodes (22 (C),(D),(F)) which on the other hand can be associated with the pattern of the whole community. They also show a strong intensity distribution at the border of the tissue. We can suppose that similarity in this border is strong enough to associate the the node in Figure 22 (E) with the green community instead of the orange one.

S13. Projection Maps for: Principal components Analysis (PCA), Non-negative matrix factorization (NMF) and Latent Dirichlet allocation (LDA)

Another common approach than the presented one to analyse the spatial distribution of imaging data is to employ dimension reduction techniques. The data set of dimension N is reduced to a dimension $k << N$, e.g. $k = 3$. Each of those dimensions show a specific distribution. The characteristics of those distributions is strongly influenced by the optimization criterions of the dimension reduction method. An overview of important properties for the applied context of this work is given in Table 1.

Each channel can assigned to one color, e.g. in RGB. The pixels of the resulting color image will be colored according to their intensity values in their respective channel. In the following examples each color channel is normalized on its own, meaning that the intensity value at the 99-percentile of each channel

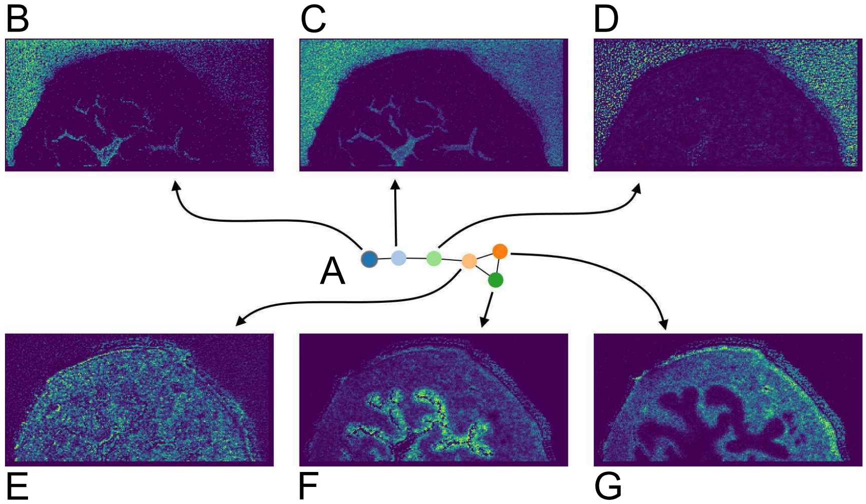

Figure 21: (A) community-graph as shown in 20, excluding all (1)–Communities. Each community-node is associated with its respective average community-map (B)-(G). For all maps viridis is used as color map.

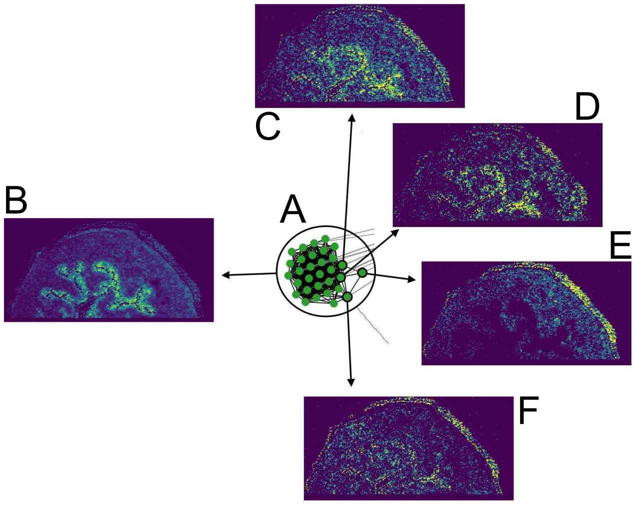

Figure 22: Questionable community assignment, revealed by network structure. (A) Community-subgraph. (B) Average community-map. (C),(D),(F) Intensity-maps of single nodes (m/z-values). (E) Intensity-map of the outlier revealed by the graph structure. It is obvious that the main intensity distribution in the middle of the tissue is missing.

is used as maximal color strength.

Examples for three common dimension reduction techniques (PCA, NMF and LDA) are shown in Figure 23 to 37.

Figure 23, 24, 25 show the first ten PCA components (four for D_G) and the projection of components one to three and two to four on the RGB color space, for data set D_G, D_B and D_T, respectively. The PCA compontents are ordered by the amount of variance they explain, i.e. their importance. For a projection into a three dimensional space it is always advisable to analyse the first four components. The reason is that the first component is often susceptible for showing either simple relations (e.g. background and foreground) or artifacts (e.g. border and interior regions).

Figure 26 to 31 show the results of NMF. NMF does not have an implicit ordering like PCA. Therefore we show both, a dimension reduction to three dimensions and the RGB projection (Figure 26, 27, 28) and a dimension reduction to ten dimensions (four for D_G) (Figure 29, 30, 31).

Figure 32 to 37 show the results of LDA. LDA is used because it is a generalization of pLSA (probabilistic latent semantic analysis), which was already analysed before (Hanselmann *et al.* (2008)). Like NMF, LDA has no implicit ordering, therefore the analysis is executed in the same way like we did for NMF. The dimension reduction to three dimensions and the RGB projection can be seen in Figure 32, 33, 34 for D_G, D_B and D_T, respectively. The dimension reduction to ten dimensions (four for D_G) is shown in Figure 35, 36, 37 for D_G, D_B and D_T, respectively.

It can be seen that the RGB projections of all three methods do well in revealing coarse distinct regions. In comparison, LDA projections provide regions with the highest contrast for D_B and D_T, followed by NMF. For D_G the highest contrast result is generated by NMF, followed by LDA. PCA projections provide the lowest contrast results in all cases. A look towards the ten component results shows that many NMF and LDA components show much more specific pattern than most of the PCA components (D_B, D_T). Comparing NMF and LDA shows that some components are quite similar between both methods. In D_B it can also be seen that the regions of those components are intensity wise more prominent for LDA. This is most likely due to the fact that D_B is a well structured sample, due to its physiology. We can conclude that for our three tested data sets LDA and NMF are more effective than PCA and at least for D_B LDA is more effective than NMF. Compared to our method fine regions like the scutellum in D_B are not revealed.

Additionally, all three methods share two problems: 1. The number of dimensions have to be predefined. Finding the best number of dimensions is a non trivial task and especially important for NMF and LDA. A comparison of figure pairs: 26 − 29, 27 − 30, 28 − 31, 32 − 35, 33 − 36 and 34 − 37 shows that the number of dimensions influences the lateral distribution of the resulting components for NMF and LDA. 2. Compared to our clustering approach it is much harder to analyse which molecules participate in which lateral distributions. This is because the components of all three methods consist of combinations of the original data samples.

Table 1: Summary of properties of three dimension reduction methods. The mentioned properties are important in the applied context.

Method	Independent of number of components	Negative values possible	High contrast projections	Implicit ordering
PCA	✓	✓	✗	✓
NMF	✗	✗	✓	✗
LDA	✗	✗	✓	✗

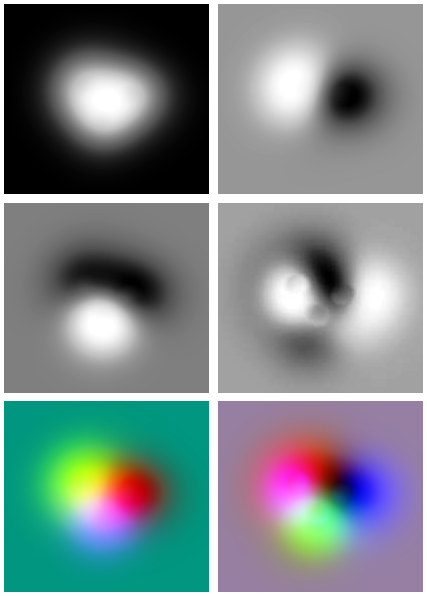

Figure 23: PCA components 1-4 of D_G, projection of component one to three into RGB space and projection of component two to four into RGB space (top-left to bottom-right) after reduction to three dimensions. Components are ordered by their importance (variance explained).

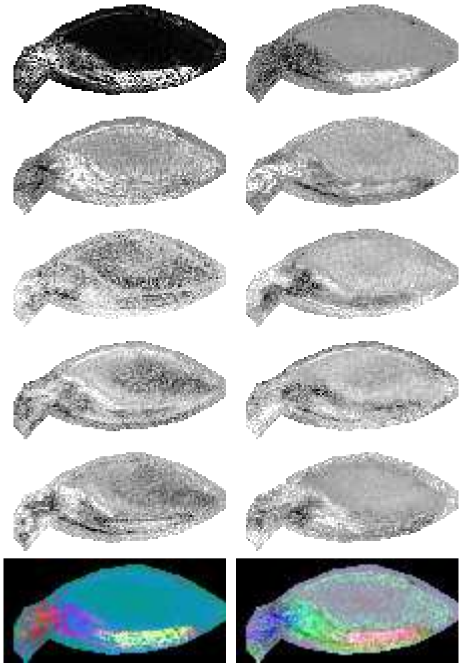

Figure 24: PCA components 1-10 of D_B, projection of component one to three into RGB space and projection of component two to four into RGB space (top-left to bottom-right) after reduction to three dimensions. Components are ordered by their importance (variance explained).

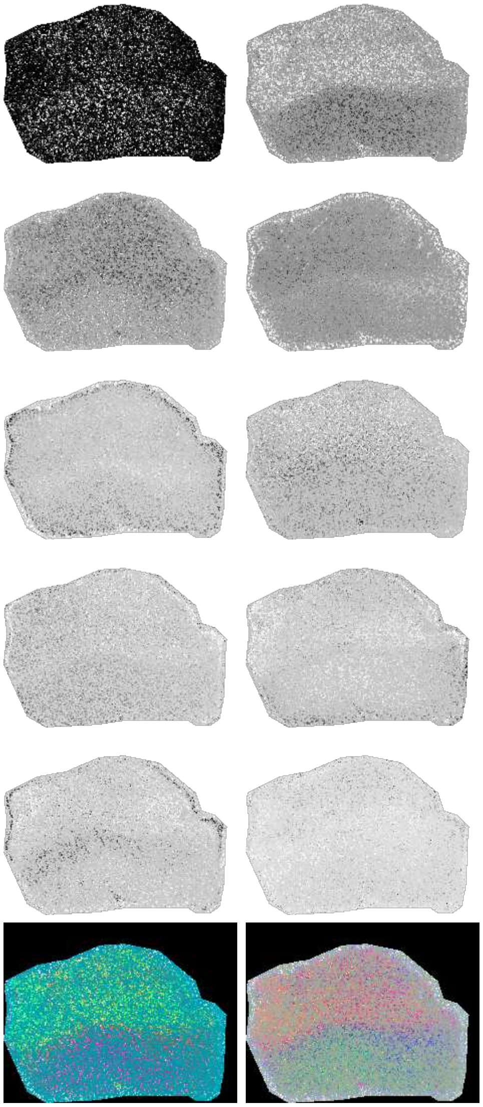

Figure 25: PCA components 1-10 of D_T, projection of component one to three into RGB space and projection of component two to four into RGB space (top-left to bottom-right) after reduction to three dimensions. Components are ordered by their importance (variance explained).

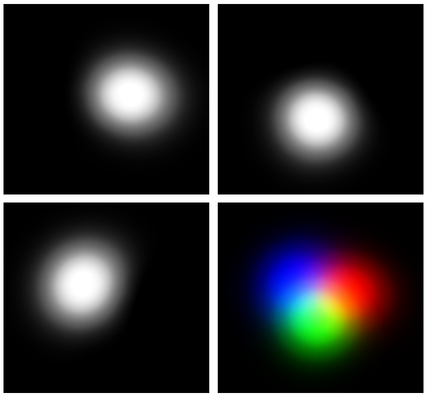

Figure 26: NMF components 1-3 of D_G and projection into RGB space (top-left to bottom-right) after reduction to three dimensions.

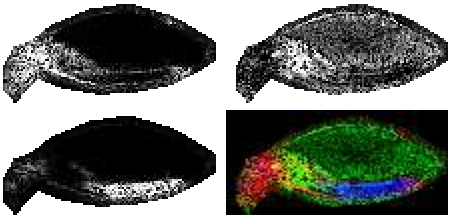

Figure 27: NMF components 1-3 of D_B and projection into RGB space (top-left to bottom-right) after reduction to three dimensions.

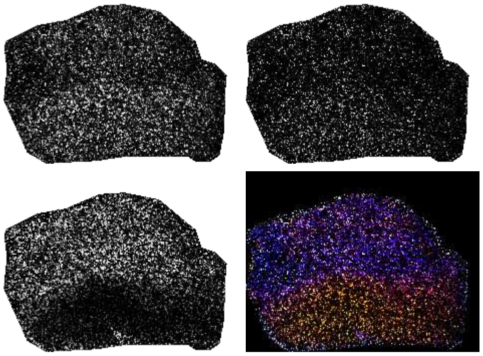

Figure 28: NMF components 1-3 of D_T and projection into RGB space (top-left to bottom-right) after reduction to three dimensions.

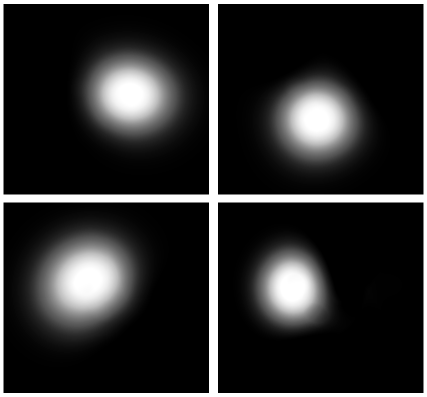

Figure 29: NMF components 1-4 of D_G (top-left to bottom-right) after reduction to four dimensions.

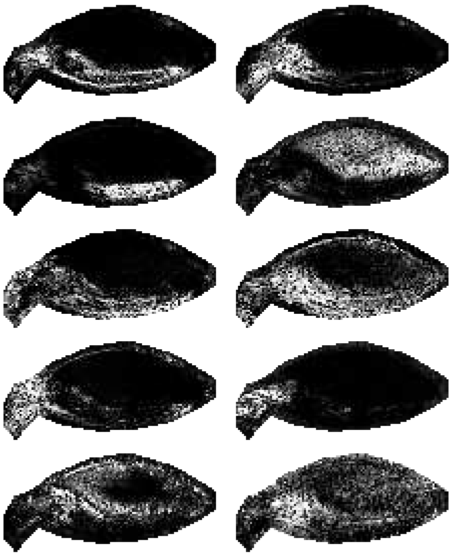

Figure 30: NMF components 1-10 of D_B (top-left to bottom-right) after reduction to ten dimensions.

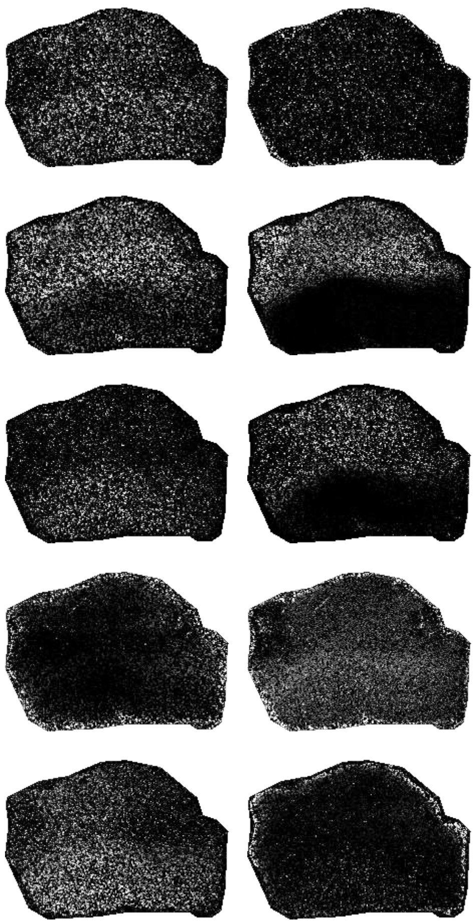

Figure 31: NMF components 1-10 of D_T (top-left to bottom-right) after reduction to ten dimensions.

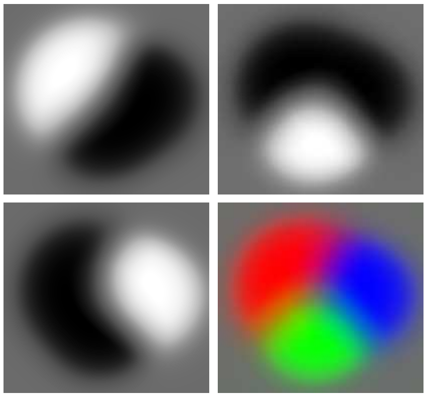

Figure 32: LDA components 1-3 of D_G and projection into RGB space (top-left to bottom-right) after reduction to three dimensions.

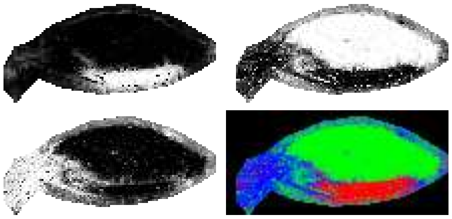

Figure 33: LDA components 1-3 of D_B and projection into RGB space (top-left to bottom-right) after reduction to three dimensions.

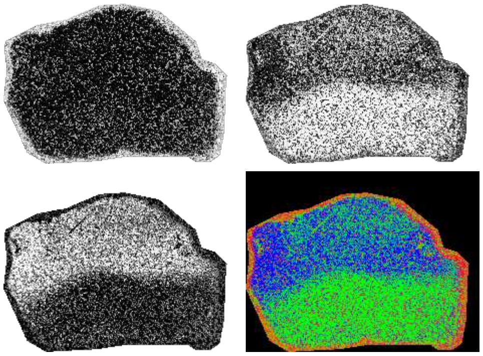

Figure 34: LDA components 1-3 of D_T and projection into RGB space (top-left to bottom-right) after reduction to three dimensions.

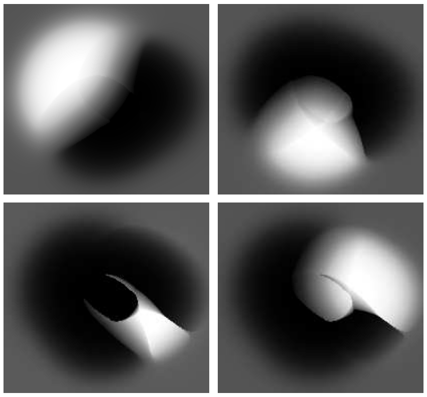

Figure 35: LDA components 1-4 of D_G (top-left to bottom-right) after reduction to four dimensions.

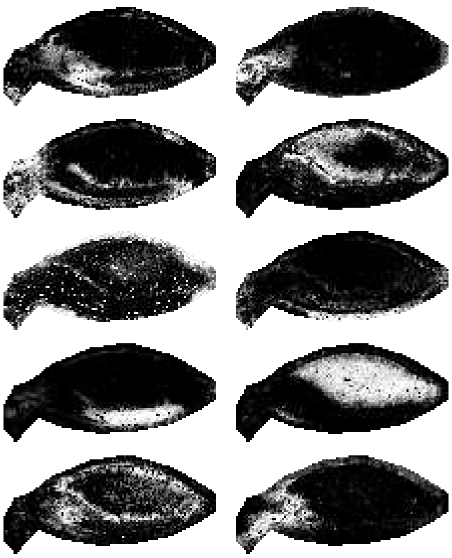

Figure 36: LDA components 1-10 of D_B (top-left to bottom-right) after reduction to ten dimensions.

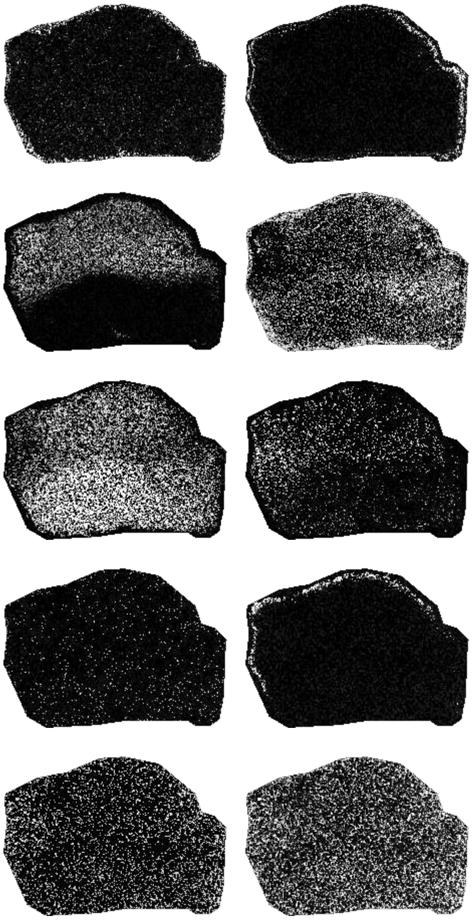

Figure 37: LDA components 1-10 of D_T (top-left to bottom-right) after reduction to ten dimensions.

S14. Implementation Details

Step one to four of our analysis pipeline is implemented in python. This includes: preprocessing, similarity calculation, threshold calculation and community detection. Currently, the source data needs to be in a special HDF5 format. This format requires a multiindex, labeled `grid_x` and `grid_y`, which describes the (x,y) pixel positions. Columns are labeled by m/z-value. This part of the analysis pipeline needs to be executed locally by calling the respective python script (`main.py` which can be found at `https://github.com/Kawue/imaging_communitydetection_demo`). The result will be a JSON file, which can be used in Grine.

Grine itself is implemented with plain Javascript, HTML and CSS. It can be started locally in a localhost. As it is currently only used for research purposes, the adaption to the own data requires code changes. One have to create an `index.html` where the `var dataPath` needs to point to the local JSON file and the `var msiImagePath` needs to point to a folder with all m/z-image png files. This folder also needs to contain a three component PCA image named as `pca_rgb.png`.

Further development to polish Grine to a final user friendly state is part of our future work.

S15. Computation Time Complexity

Regarding the computation time three main parts need to be considered: 1. Calculation of the similarity matrix, 2. threshold selection to transform the similarity matrix to an adjacency matrix and 3. community detection.

Pearsons correlation coefficient can be computed in linear time.

The transformation method is more complex as it needs to compute a PCA and utilizes graph properties. The used SVD based implementation has a complexity of $\mathcal{O}(n_{\max}^2 n_{\min})$, where $n_{\max} = \max(n_{\text{samples}}, n_{\text{features}})$ and $n_{\min} = \min(n_{\text{samples}}, n_{\text{features}})$. The average clustering coefficient on sparse graphs has a complexity of $\mathcal{O}(v^2)$, while the global efficiency has a complexity of $\mathcal{O}(p(e + vlogv))$, where p, e, v is the number of all possible vertex pairs, edges and vertices, respectively. Considering the fact that we perform those calculations for a series of candidate graphs, where the series is of length t, this leads to a total complexity of $\mathcal{O}(t((p(e + vlogv)) + v^2) + n_{\max}^2 n_{\min})$. Since $n_{\max}$ equals t and $n_{\min}$ equals two we can simplify to $\mathcal{O}(t(p(e + vlogv)) + tv^2 + 2t^2)$. We can also rewrite p as $\frac{v!}{2!(v-2)!}$ leading to $\mathcal{O}(t(\frac{v!}{2!(v-2)!}(e + vlogv)) + tv^2 + 2t^2)$. Since t is a freely choosable parameter this expression is clearly dominated by $\frac{v!}{2!(v-2)!}$, i.e. the number of nodes.

The computation time of the leading eigenvector community detection for a sparse graph is $\mathcal{O}(v^2 \log v)$, where v is the number of vertices.

We would like to mention that some of these complexities rely on estimations from either papers or workshops. Therefore, no proof may be available and we cannot guarantee their correctness. However, this analysis should provide a good impression for complexity and performance.

Without going into more detail here it is obvious that both, the transformation and the community calculation complexity depends on the number of vertices. Meaning, that the number of m/z-values, i.e. vertices, limits our method with factorial complexity. Ignoring its mediocre results, we could use a linear transformation method. This can be achieved by relying on simple statistics like mean and standard deviation. However, the number of vertices would still limit the method with a factor of $v^2 \log v$. Moreover, too many vertices can also cause clutter problems in our visualization.

On the other hand the size of the m/z-images is only important for the pearson correlation coefficient calculation. Due to the linear complexity the image size does not limit our analysis significantly.

To make our method applicable to data sets with a high amount of peaks the the transformation method could be changed to a less complex variant, e.g. relying on statistics and the community algorithm could be changed to a greedy one. Those changes would improve the performance a lot but also result in a decreasing network and community quality.

S16. Workflow Dependencies for Parameters and Functions

During this study we had to make three main methodological decisions, i.e. 1. which similarity measure, 2. which thresholding method and 3. which community detection algorithm. In this part we explain how these three most important steps influence each other and their impact on the downstream MSI analysis. Considered alternatives will be discussed in section S17.

The explanation will be backwards to facilitate understanding.

The quality and characteristics of the detected communities depends on the algorithms optimization criterion. Many community detection algorithms try to optimize criteria like minimizing inner group distances and maximizing inter group distances. The result quality of the community detection algorithm depends strongly on the graph structure. A graph with strongly pronounced group structures will provide better results than one with weakly pronounced group structures. The graph structure itself strongly depends on the calculated threshold value, as this determines presence and absence of edges. For this reason, we use a computationally expensive method, which is based on criteria that are closely connected to the optimization strategies of different community detection algorithms. Lastly, the threshold detection works best if the computed similarity values are not evenly distributed and cover a broad range. This allows an easier distinction between similar and dissimilar.

In summary it can be said that the descriptive ability of the similarity measure and the threshold selection are the most critical points in our analysis workflow. The selection of the community detection algorithm influences the characteristics of communities, depending on the optimization criterion. Also, different algorithms provide different features, like overlaps, hierarchies or membership strength.

It is obvious that every step has a strong influence on the downstream analysis. Choices for the similarity measure and the threshold selection can be made independent of the research question. Choices for the community detection algorithm may differ depending on provided features.

Small-worldness is often considered as a structure that is suitable to describe relationships on biological graphs. Therefore we made most of our decisions with this principle in mind. Out of all tested similarity measures the pearson correlation had the broadest value range and was suited for the high variability in MALDI-MSI data. The threshold selection method was developed to reward dense groups and solid inter group connectivity, which is the quintessence behind small-worldness. As the community detection algorithm, we chose the leading eigenvector method for a couple of reasons. First, it has a well traceable optimization criterion (modularity). Second, it has a automatic stop criterion, i.e. it does not need an apriori number of communities. Third, in the method section we explained that the community assignment is based on the sign of the elements in the leading eigenvector. While the magnitude of the elements is currently ignored there is still information in those values. In fact, the magnitude describes how strongly a node belongs to a group (Newman (2006)). This allows for two possible future enhancements. First, ambiguous community assignments can be spotted and resolved. Second, this information could be included in Grine to allow for a more in depth analysis regarding the community structure.

S17. Alternatives

Similarity Measure:
During this study we computed communities with different measures. We chose the pearson correlation as it produced consistently good results with a low computation time. Cosine similarity and mutual information were considered as alternatives. However, pearson correlation produced better results. We assume it is slightly better suited than cosine similarity because our data is not mean centered and has a non negligible amount of instrument induced variation. Mutual information seems to have problems with the high pixel to pixel variation inherent to MALDI-MSI and is therefore not suitable.

Threshold Calculation:
We began to calculate the threshold based on very basic statistics, i.e. $mean(S) + c * std(S)$, where S is the set of all similarity values and c is a constant. The results were very unstable. Next, we tried the approach from Zahoránszky-Kőhalmi *et al.* (2016), which chooses the threshold based on the average clustering coefficient. While the results were more stable we found synthetic examples that produced bad results. We concluded that the average clustering coefficient alone does not provide enough information about the network. With the small-worldness criterion in mind our current implementation was developed. This method produces the most stable results and also works on the synthetic examples.

Community Detection:
A few alternatives for the community detection algorithm were considered.

We tried an overlapping community detection approach called **clique percolation method** Palla *et al.* (2005). To explain our decision to work with spatial distributions, we argued that the assumption of existing overlapping information is a more realistic setting in biology. This also counts for overlapping community structures. However, their computation is harder and the analysis is more complex. As our approach is novel for the MSI community we decided to focus on non-overlapping community structures. Also, the current implementation of Grine is not capable to handle overlapping communities.

We also considered two non-overlapping community methods. The first one is called **Louvain Method** (Blondel *et al.* (2008)). While the exact complexity of this method is not known it seems to run in $\mathcal{O}(v \log v)$, where v is the number of vertices. The method allows to create a hierarchical community structure. However, at the current time Grine is not able to handle hierarchical community structures. On the evaluated data sets, the Louvain Method and the leading eigenvector method worked approximately equally well. As long as Grine is not capable to deal with hierarchical communities, we value the possibility to resolve ambiguities higher. The second method, called **fluid communities** (Parés *et al.* (2017)), did not provide an appropriated quality.

There are a lot of different state-of-the-art community detection methods. An extensive analysis on their differences and impact on MSI data would be a study on its own and seems like an interesting topic for future work.

S18. Limitations and Considerations

The assumption of spatially bound metabolic activity is a limitation that targets the definition of similarity. Extending our definition of similarity in a way that makes it location independent would make this restrictive assumption obsolete. One could consider to either change the m/z-images by extensive transformations like scaling, rotation and alignment or to change the definition of similarity directly. However, both variants have their own complications. Extensive transformations are likely to yield a not negligible number of false positive relationships. Additionally, the inherent variability in distribution shapes and intensity values makes this problem even harder. Changing the similarity measure itself is an even more complex problem. It requires an initial comparative study of existing methods, followed by introducing changes into existing measures or the creation of new ones. Because of its vast scope this problem requires a study of its own and is one of our future research interests.

In this work we were interested in similar and locally bounded distributions. Two other interesting questions arise that are closely connected to this problem: 1. Inverse localization and 2. shared regions. Both questions cannot be answered right now but are interesting topics for future research. While not going into detail, we believe that the graph is an appropriate tool to investigate both questions. If the similarity measure is suitable, inversely located regions should be far away in the graph, i.e. many hops are needed to connect them. On

the other hand, communities that share regions, like common borders, should be very closely connected. If these assumptions hold, Grine could be extended to automatically find sets of candidates for both cases.
